# Unmasking chloride attack on the passive film of metals

**DOI:** 10.1038/s41467-018-04942-x

**Published:** 2018-07-02

**Authors:** B. Zhang, J. Wang, B. Wu, X. W. Guo, Y. J. Wang, D. Chen, Y. C. Zhang, K. Du, E. E. Oguzie, X. L. Ma

**Affiliations:** 1 Shenyang National Laboratory for Materials Science, Institute of Metal Research, Chinese Academy of Sciences, Wenhua Road 72, 110016 Shenyang, China; 2 Electrochemistry and Materials Science Research Laboratory, Department of Chemistry, Federal University of Technology Owerri, PMB, Owerri, 1526 Nigeria; 3 State Key Lab of Advanced Processing and Recycling on Non-ferrous Metals, Lanzhou University of Technology, 730050 Lanzhou, China

## Abstract

Nanometer-thick passive films on metals usually impart remarkable resistance to general corrosion but are susceptible to localized attack in certain aggressive media, leading to material failure with pronounced adverse economic and safety consequences. Over the past decades, several classic theories have been proposed and accepted, based on hypotheses and theoretical models, and oftentimes, not sufficiently nor directly corroborated by experimental evidence. Here we show experimental results on the structure of the passive film formed on a FeCr_15_Ni_15_ single crystal in chloride-free and chloride-containing media. We use aberration-corrected transmission electron microscopy to directly capture the chloride ion accumulation at the metal/film interface, lattice expansion on the metal side, undulations at the interface, and structural inhomogeneity on the film side, most of which had previously been rejected by existing models. This work unmasks, at the atomic scale, the mechanism of chloride-induced passivity breakdown that is known to occur in various metallic materials.

## Abstract (web summary)

Collecting experimental evidence of chloride ion attack on protective passive metallic films due to corrosion remains challenging. Here, the authors show that the boundaries between nanocrystals and amorphous regions in the passive film ease chloride transport even as they do not coincide with areas of high chloride concentration.

## Introduction

Corrosion is one of the major causes of material failure and hence leads to a huge cost to our society^1^. The nanometer-thick passive film on metals resists a general corrosion, but it is susceptible to severe localized attack in certain aggressive media^2^. The best-known inducer of localized passive film breakdown is the chloride ion. Despite the enormous amount of experimental data and diverse hypotheses and models proposed till date^1–13^, the breakdown of the passive film is still not sufficiently understood and remains one of the most important and basic problems in corrosion science.

The lack of agreement on the mechanism of passive film breakdown is mainly due to the difficulty encountered in obtaining precise experimental information. To clarify the exact nature of the chloride-induced breakdown, Cl^−^ incorporation to the film has to be experimentally confirmed and the accurate location needs to be identified. In the meanwhile, chloride-induced modification to the film has to be experimentally addressed as well. It is worthy of note that these issues were extensively studied by X-ray photoelectron spectroscopy (XPS)^7,14–21^, Auger electron spectroscopy (AES)^7,14,16,19,22–27^, secondary ion mass spectrometry^7,8,16,19,25^, and radiotracer techniques^11^. Nonetheless, it is still very difficult and challenging to guarantee the precision and accuracy of observed locations and concentrations of a very small amount of chloride in an extremely thin passive film with a thickness of only a few nanometers. Much of evidence on the incorporation of Cl^−^ in the passive oxide film can be mainly classified into two groups: one is chloride incorporation^7,8,11,14–17,22–24^, and the other is chloride absence in the passive film^14,18–21,25–27^. In the case of incorporation, the location of chloride in the passive film is also controversy. Some investigators declare that Cl^−^ locate or concentrate in the outer layer of the film^7,14–16,22,23^, whereas some others claim it is in the inner layer^8,24^. In reality, most of the reported methods do not directly identify the presence of chloride or its location within the passive film. Alongside questions regarding chloride ion distribution in the passive film are issues relating to the nature of atomic-scale interactions between the passive film and chloride, i.e., issues regarding how, where, and when the chloride modifies the passive film. To the best of our knowledge, although the experimental advances, including transmission electron microscopic (TEM) characterization^28–31^, promote powerful evidence on the structure and chemistry of the passive film^20,28,29,31–45^ as well as the evolution imparted by chloride^36,39,43,46^, no technique has succeeded in directly following the evolution of the passive film, in chloride-containing media, across the entire film ranging from the surface to the metal/film interface.

Without doubt, deciphering the interactions of the chloride ion with the passive film, including chloride-induced modifications to the properties of the passive film at the atomic scale, is key to understanding the precise mechanism of passivity breakdown. In the present work, using aberration-corrected TEM (Cs-corrected TEM) and a fast and precise super X-ray energy-dispersive spectrometer (Super-X EDS) analysis with four detectors, we simultaneously investigate the film and metal matrix as well as their interface via cross-sectioning in real space. We find the chloride accumulation within the inner layer of the passive film and the associated fluctuations at the matrix/passive film interface. We provide direct evidence on the location of chloride and the resultant phenomena of the lattice expansion on the metal side, undulations at the interface, and structural inhomogeneity on the film side. The present findings allow for the atomic-scale mechanism of passivity breakdown to be revisited on the basis of real-space imaging in multi-dimensions.

## Results

### Sample preparation under various chemical conditions

Passive films were formed on the (001) and (110) plane, respectively, of FeCr_15_Ni_15_ single crystal (Supplementary Note 1 and Note 2, Supplementary Figs 1–3). This enabled us to obtain a distinct metal/passive film interface (Supplementary Fig. 4) and better characterize the structure of the interface region. On the other hand, the single crystal, which is free of any inclusions and grain boundaries, yields a high-quality passive film with a continuous coverage on the alloy matrix. It also effectively avoids the paradigm of the weakest sites breaking down the soonest, which makes figuring out the intrinsic mechanism complex (Supplementary Note 2). In order to monitor the transport and effect of chloride ions, passive films were formed under three designated conditions: passivation in H_2_SO_4_ electrolyte, passivation in H_2_SO_4 _+ NaCl electrolyte, and initial passivation in H_2_SO_4_ electrolyte and subsequent addition of NaCl into the H_2_SO_4_ electrolyte (Supplementary Note 3, Supplementary Figs 5 and 6). The cross-sectional TEM specimen was prepared by the conventional method, that is, passivated surfaces of two samples were bonded face-to-face and then thinned by grinding and ion-milling. During sample preparation and subsequent TEM observation, the extremely thin passive film was strictly ensured free of mechanical and beam-induced damage (Supplementary Note 4).

### Structural evolution of passive film with aging in air

**Fig. 1:**
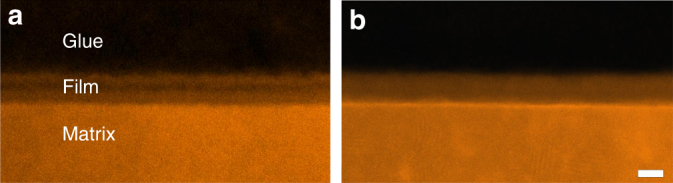
HAADF-STEM image showing the contrast evolution of the passive film on FeCr_15_Ni_15_ single crystal on exposure to air. Scale bar, 5 nm. **a** Image immediately after the specimen was prepared. **b** Image after the specimen has been exposed in air for about 6 days

**Fig. 2:**
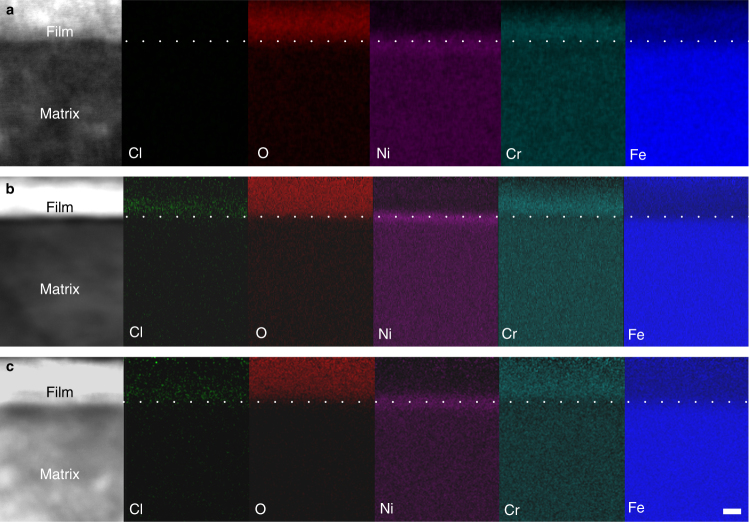
Super-X EDS mapping showing chloride ion incorporated in and penetrating the passive film and accumulating at the matrix/passive film interface. Element maps of the film formed in **a** 0.5 mol L^−1^ H_2_SO_4_ electrolyte at 640 mV/SHE for 30 min; **b** 0.5 mol L^−1^ H_2_SO_4 _+ 0.3 mol L^−1^ NaCl electrolyte at 640 mV/SHE for 30 min, and **c** passivated in 0.5 mol L^−1^ H_2_SO_4_ electrolyte at 640 mV/SHE for 30 min with subsequent addition of NaCl. All scale bars in **a**–**c** are 2 nm

The TEM observation in the high-angle annular-dark-field (HAADF) mode and Super-X EDS analysis on the passive film were performed and the results are shown in Figs. 1 and 2. Figure 1a is the HAADF scanning transmission electron microscopic (HAADF-STEM) image showing the passive film on FeCr_15_Ni_15_ single crystal formed in H_2_SO_4_ electrolyte (condition 1). According to the contrast difference, the film seems to be tri-layer structured. Whereas the EDS mapping analysis (Fig. 2a) indicates a well-defined bi-layer structure with the inner Cr-rich layer and the outer Fe-rich layer, as generally accepted^29,47–51^. Interestingly, after aging the specimen in air for a few days, we also subjected the aged specimen again to TEM observation and we found that the contrast had become homogeneous, following disappearance of the middle darker-contrast layer (Fig. 1b). The implication is that the outer layer of the passive film, formed by precipitation of the hydrolyzed metal cations, is transformed to the metal oxide by a dehydration reaction, which was confirmed by XPS analysis^34,38,39,50,51^. It is worthwhile to mention that the HAADF mode image provides an incoherent image using high-angle scattered electrons, where the contrast is strongly dependent on the scattering ability of heavy atoms. Thus the much denser metal matrix would show the brightest contrast, followed by the metal oxide, with metal hydroxide displaying the darkest contrast. From the foregoing and taking into consideration of the EDS mapping results, the inner layer is a Cr-rich oxide, while the outer layer, with the darkest contrast should be Fe-rich hydroxides. The unavoidable exposure in air going from passivation in the electrolyte to TEM observation allows the outmost hydroxide layer to be partially dehydrated yielding the brighter oxide, thus giving the film an apparent tri-layered structure in the HAADF-STEM image. With prolonged exposure, the hydroxide layer became completely dehydrated and the passive film correspondingly reverted to an oxide film. Although the oxide film has a bi-layered structure distinguished from the Cr-rich and Fe-rich layer (Fig. 2a), it can be hardly identified in the image taken with the HAADF mode (Fig. 1b), since the contrast in the HAADF image is strongly dependent on the scattering ability of heavy atoms that is associated with the atomic number. Here the atomic number of Fe and Cr is rather close, making the contrast of Cr-rich oxide layer and Fe-rich oxide layer seems homogeneous. The above results provide direct experiment evidence of a dehydration model.

### Structural evolution of the interface imparted by chloride

**Fig. 3:**
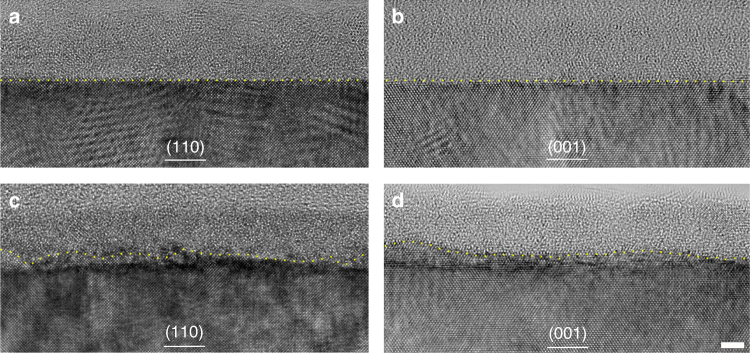
Chloride-induced undulations at the metal/passive film interface, wherein the yellow line indicates the metal/passive film interface. Scale bar, 2 nm. **a**, **b** HRTEM images along the [001] and [110] axes of the austenitic matrix showing the passive film grown on (110) and (001) planes of FeCr_15_Ni_15_ single crystal in 0.5 mol L^−1^ H_2_SO_4_ electrolyte, revealing a sharp and straight interface, even at the atomic scale. **c** HRTEM images along [001] axis showing the passive film grown on (110) plane in 0.3 mol L^−1^ NaCl + 0.5 mol L^−1^ H_2_SO_4_ electrolyte, revealing an indistinct and substantially undulating interface. **d** HRTEM images along [110] axis showing the passive film initially grown in 0.5 mol L^−1^ H_2_SO_4_ electrolyte for 30 min with subsequent addition of NaCl into the H_2_SO_4_ electrolyte. The interface is as well indistinct and substantially undulating

Prior to studying the interactions of chloride ions with the passive film and austenitic matrix, the pristine passive film formed in chloride-free electrolyte was analyzed, in which attention was paid to the structure and the element distribution within the film and near the interfaces. Cs-corrected TEM revealed a passive film with thickness of about 4–5 nm (see TEM image in Supplementary Fig. 4). The corresponding EDS maps are shown in Fig. 2a. A well-defined bi-layer passive film is seen, where the inner (barrier) layer adjacent to the metal is enriched in Cr and depleted in Fe, while the outer (precipitated) layer is depleted in Cr and enriched in Fe. It is noteworthy that the film/matrix interface is sharp, well defined, and straight, even at the atomic level. On the matrix side, immediately adjacent to the passive film/matrix interface there exists a layer with Cr depletion and Ni enrichment, which is in agreement with the previous findings^29,31,48,51^. Cross-sectional high-resolution TEM (HRTEM) images, obtained along the [001] and [110] axes (Fig. 3a, b) of the austenitic matrix, respectively, show that the passive film is mostly amorphous.

The passive film formed in chloride-containing H_2_SO_4_ solution (condition 2) is shown in Fig. 3c. It is immediately obvious that the previously sharp and straight interface in chloride-free solution has become indistinct and substantially undulating. This is indisputably a consequence of chloride ion attack. By means of Super-X EDS mapping experiments, we successfully obtained the distribution of elemental Cl in the passive film, as shown in Fig. 2b, which, interestingly, shows Cl to be concentrated within the inner layer, (average content ~1 atomic %). Supplementary Figure 7 also shows a distinctive Cl peak in the composition spectrum. Significantly lower amounts of Cl (0.1 atomic %) were detected in the outer layer. Evidently, these findings indicate that the chloride ions incorporate in the passive film, permeate the outer and inner layers, and ultimately attack the interface, giving rise to the undulating interfacial structure. Such an incorporation of solution species in the inner layer has not been previously observed and is actually considered non-feasible by some existing theories on passivity breakdown^5,10,13,14,52^.

We designed another experimental procedure, wherein the FeCr_15_Ni_15_ single crystal was initially passivated in H_2_SO_4_ electrolyte for 30 min and subsequently NaCl was added into the H_2_SO_4_ electrolyte (condition 3). This enabled us to monitor the attack of chloride ions on the as-grown passive film. By careful examination of a series of interfaces, what we observed is a reduced prevalence of the undulating interface, which only manifested at a few locations (Fig. 3d). These locations are expected to be terminal points for paths through which chloride ions permeate. Correspondingly and maybe not unexpectedly, Cl was only detected at locations manifesting the undulating interfaces, typically like that in Fig. 2c and was not detected (Supplementary Fig. 8) at the still distinct and unperturbed interfaces (similar to that in Fig. 2a). It is thus obvious that chloride ions only get to certain interfacial locations by heterogeneously penetrating the as-grown film.

### Nanocrystal-amorphous interface assisting Cl^−^ permeation

**Fig. 4:**
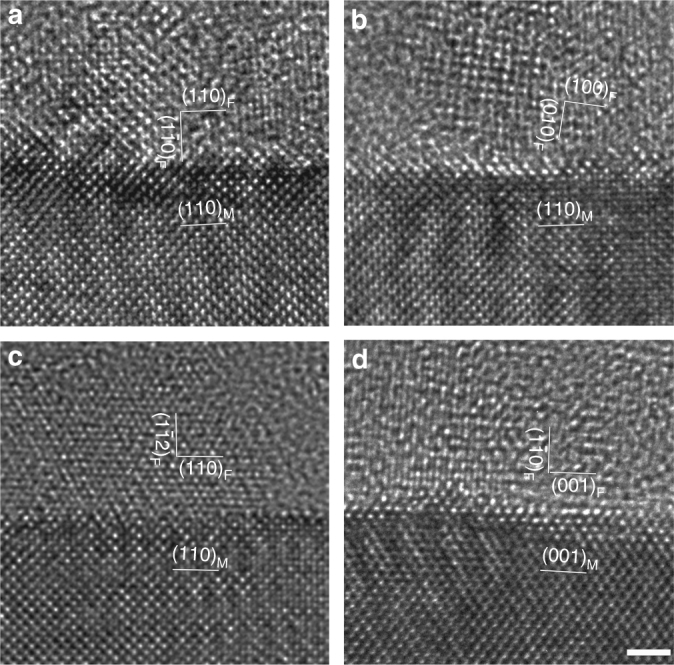
Cross-sectional HRTEM images along the [001] and [110] axes of the austenitic matrix showing the interface between the passive film and steel matrix. It is seen that the passive film is mainly amorphous, with some nanocrystals. A series of HRTEM images obtained from variant orientations and locations indicate that the nanocrystals feature face-centered cubic structure. In most cases, the nanocrystals have crystallographic orientations with austenitic matrix but occasionally not. This figure displays typical configurations where the orientation relationships between the nanocrystals in the passive film and the single-crystalline substrate are labeled. **a** Film (110) || FeCr_15_Ni_15_ (110) and film (1–10) || FeCr_15_Ni_15_ (1–10). **b** The crystal is randomly oriented with no orientation relationship with the austenitic matrix. **c** Film (110) || FeCr_15_Ni_15_ (110) and film (1–10) || FeCr_15_Ni_15_ (1–12). **d** Film (001) || FeCr_15_Ni_15_ (001) and film (1–10) || FeCr_15_Ni_15_ (1–10). Scale bar, 1 nm

It is well known that grain boundaries exhibit an irregular atom array yielding a loose structure that usually provide tunnels for species diffusion and transport. In contrast, the amorphous phase always features a random atom distribution, so the diffusion and transport processes in amorphous materials are not yet well understood. It is noteworthy that, although the passive films in the present study are mostly amorphous as seen in the HRTEM images, some periodic two-dimensional lattices with a scale of 1–3 nm are often visualized, that is to say, a small amount of nanocrystals (NCs) are embedded in the passive films. Figure 4a–d display typical cross-sectional HRTEM images in which some NCs inherently present in the amorphous passive film. A series of HRTEM images obtained from variant orientations and locations indicate that NCs feature face-centered cubic structure (also seen in Supplementary Fig. 9). In our present study, the NCs often display specific crystallographic orientation relationships with the austenitic matrix but occasionally not. Thus the grain boundary between the NCs and amorphous phase can be readily identified according to the HRTEM images. Nevertheless, a determination of atomic configurations at this kind of boundary is a challenge, which should be more complex than that of two crystalline grains. We hypothesize that the interfaces between the NCs and the amorphous zone assume the features of atomic irregularity and thus provide ready paths for chloride ion transport.

The selective permeation implied by our results arises from the inherently non-homogeneous microstructure of passive films and depends on the nature of and interconnection between the paths created along the NCs/amorphous zone interface. When a connected path traverses the entire thickness of the passive film, chloride ions tunneling through those paths would eventually arrive at the matrix/passive film interface. On the other hand, where no connected paths exist, or all the paths are abridged, the matrix/passive film interface would remain unperturbed because chloride ions are unable to get through.

**Fig. 5:**
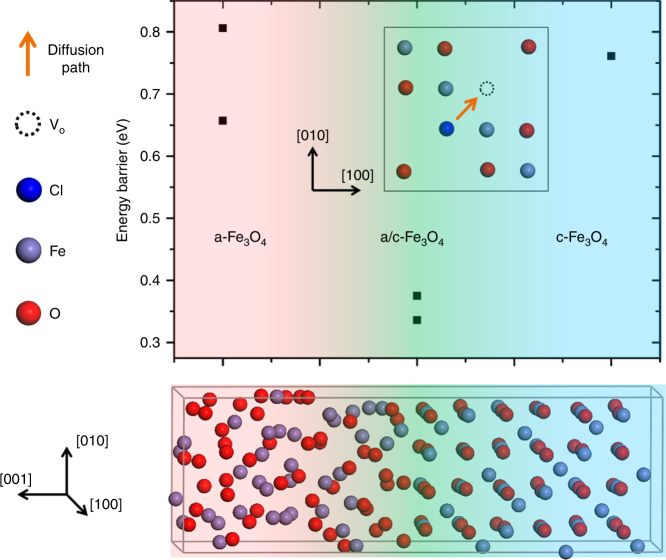
Energy barriers to Cl^−^ ion diffusion from one oxygen vacancy to a neighboring one in the three zones. Representative schematic diagram of the diffusion paths in c-Fe_3_O_4_ is inserted. The diffusion barrier is lowest at the interface region, thus confirming that the interface between the nanocrystals and the amorphous zone provides a favorable channel for chloride ion diffusion

In order to provide a theoretical basis for the above assertions, we performed first-principle computations to model the diffusion of chloride ions within the passive film. The supercells constructed for the simulation contain the NC structure, the amorphous zone, and the amorphous zone/NC interface, respectively. This enables a quantification of the diffusion barriers to chloride ion diffusion from one oxygen vacancy to the next, as illustrated in Fig. 5. Details of the computation procedure can be found in Supplementary Methods. Actually, for a qualitatively comparison, the trend of diffusion barriers in these three zones has nothing to do with the specific crystal structure. So, for simplicity in the calculations, we selected the binary spinel Fe_3_O_4_ representing the spinel-structured NC embedded in the passive film. By comparing the energy barriers for Cl^−^ ion diffusion from one oxygen vacancy to its neighboring one, our computations show the diffusion barrier to be lowest at the interface region, thus confirming that the interface between the NCs and the amorphous zone provides a favorable channel for chloride ion diffusion.

In earlier studies, several techniques, including reciprocal space identification by XRD analysis^32,53–55^, electron diffraction in a TEM^56–58^, and real-space imaging upon the outmost surface by scanning tuneling microscopic technique^33,42,50,51,59–62^, have been applied in determining structural information on the nano-crystalline nature of the passive films, and the role of grain boundaries in passivity breakdown and initiation of localized corrosion has been widely discussed^63–65^. It is worthwhile to mention that, although the grain boundary was specified to the boundary between nano-crystalline oxide grains in those works, different from the boundary between NCs and the amorphous zone we propose, it is strongly implied that full amorphization has greater tendency to resist localized attack due to the absence of the ready path (grain boundaries) for outward migration of cations or inward migration of anions^60,66–68^.

### Interface strain distribution modeling

**Fig. 6:**
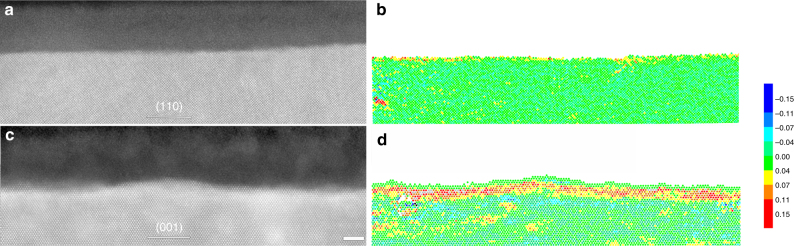
LADIA simulation results showing the strain state in the matrix side near the metal/passive film interface. Elastic strain component measured from HAADF-STEM image intensity peaks (normal to the interface plane between the passive thin film and the alloy specimen) through the alloy crystal. Scale bar, 2 nm. **a** High-resolution HAADF-STEM image along the [001] direction of the austenitic matrix showing the passive film on (110) of FeCr_15_Ni_15_ single crystal in 0.5 mol L^−1^ H_2_SO_4_. The interface is sharp and straight at the atomic scale. **b** LADIA simulation map based on **a**, which shows no evidence of lattice expansion and associated tension. The image shows the local expansion/contraction of next-neighbor atom column distances in the alloy along the [110] direction. **c** High-resolution HAADF-STEM image along the [110] axis showing a passive film formed in 0.5 mol L^−1^ H_2_SO_4 _+ 0.3 mol L^−1^ NaCl electrolyte, with corresponding undulating interface. **d** LADIA simulating map based on **c**, revealing obvious lattice expansion, with associated induced tension. The image shows the local expansion/contraction of next-neighbor atom column distances in the alloy along the [100] direction. The color bar on the right indicates the normal strain, where colors for positive values represent tensile strain and colors for negative values represent compressive strain

The as-prepared single crystal matrix provides us an opportunity to examine the strain state within the matrix side using a well-established LADIA simulation method based on the high-resolution HAADF-STEM images, since the local lattice distortion immediately below the interface would be induced if a stress exists at the interface. The strain state within the matrix side corresponding to the two types of metal/film interfaces visualized in our study (i.e., straight and undulating interfaces) was modeled using the LADIA simulation method (Supplementary Methods). The results are shown in Fig. 6 and Supplementary Fig. 10. The straight interface formed in chloride-free electrolyte revealed no signs of lattice distortion on the matrix side (Fig. 6a, b). Conversely, the undulating interface formed in chloride-containing electrolyte shows clear evidence of strain-induced lattice expansion on the matrix side (Fig. 6c, d). The local strain state that we have extracted is along the direction parallel to the interface of metal/oxide and meanwhile perpendicular to the viewing direction (Supplementary Figure 11a and 11b). It is proposed that the strain state along the viewing direction is also the case, since these two perpendicular directions are crystallographically equivalent, as shown in Supplementary Fig. 11c. So the local strain state is in two dimensions parallel to the metal/film interface. The lattice expansion in the matrix side means a tensile strain at the interface. Correspondingly, a pronounced tension is imparted to the passive film as a result of interface undulating induced by chloride ions.

### Chloride-induced inhomogeneity to the passive film

According to the image contrast in Fig. 6 and Supplementary Fig. 10, the passive film formed in chloride-free solution appears quite uniform and compact, going from the homogeneous contrast of the HAADF-STEM image. In contrast, the inhomogeneous contrast in the film obtained in chloride-containing solution corresponds to a non-uniform and rather loose passive film, which is indisputably resultant from the chloride ion incorporation to the passive film. Especially at the inner layer, the image contrast is much darker, which indicates that the film is much looser (note that the image is taken in the HAADF mode, and the contrast is strongly associated with the mass density of heavy atoms). Based on a combination of HAADF imaging and EDS analysis of chloride concentration to the inner layer aforementioned, we propose that some Me_3_(OCl)_*n*_ species might be formed at the inner layer of the passive film in chloride-containing environments.

## Discussion

**Fig. 7:**
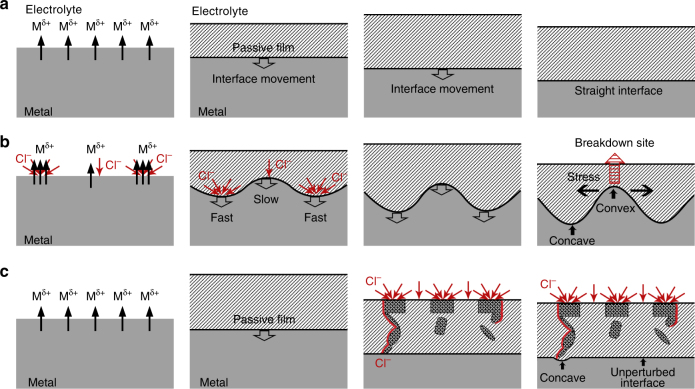
Schematic maps illustrating interface evolution in the absence and presence of chloride ions. The film growth process involves transport of both injected metal ions from the matrix and oxygen in solution through the barrier layer, causing the metal/film (Me/BL) interface to move toward the metal matrix side. **a** In chloride-free (0.5 mol L^−1^ H_2_SO_4_) electrolyte, the austenitic matrix experienced selective dissolution of metal ions (major Fe) in a somewhat homogeneous manner, yielding a straight Me/BL interface at the atomic scale. **b** In chloride-containing (0.5 mol L^−1^ H_2_SO_4 _+ 0.3 mol L^−1^ NaCl) electrolyte, the tendency of chloride ions to be preferentially adsorbed at defective sites yields non-homogeneous adsorption on the bare metal surface. High chloride ion concentration would induce faster dissolution of Fe, which leads to inhomogeneous interface-movement rates, hence differences in passive film growth rates. Such a process yields a passive film with an irregular and undulating Me/BL interface. **c** When chloride ions attack the as-grown passive film, chloride ions only get to certain interfacial locations by heterogeneously penetrating the as-grown film along the connected path provided by the interfaces between nanocrystals and the amorphous zone. This gives rise to an undulating interface

In chloride-free electrolyte, the austenitic matrix experienced selective dissolution of both the major Fe and minor Cr components in a somewhat homogeneous manner, promoting initial formation of the inner Cr-rich oxides at the metal/solution (Me/Sol) interface. The dissolved Fe and Cr cations were then simultaneously hydrolyzed and subsequently re-deposited, to yield the outer Fe-rich precipitation layer, as illustrated in Fig. 7a. At this stage, the film growth process involving transport of both injected metal ions from the matrix and oxygen in solution through the barrier layer was faster than the film dissolution process, causing the metal/film (Me/BL) interface to move toward the metal matrix side. With time, dynamic equilibrium would be attained, where the velocity of film growth equaled that of film dissolution. Even though the interfaces of Me/BL can still keep moving at dynamic equilibrium, the thickness of the passive film remained more or less constant. These processes thus yielded a straight Me/BL interface at the atomic scale, as illustrated in Fig. 7a.

The propensity of chloride ions to be preferentially adsorbed at certain non-uniformly distributed distinct defective locations means that the chloride ions would be non-homogeneously adsorbed on the bare metal surface and could modify interfacial process mechanisms in several ways. For one, chloride ions particularly promote Fe and to some extent Cr dissolution by coordination:

1 $${\mathrm{Me + Cl}}^{\mathrm{ - }}{\mathrm{ = MeCl}}^{{n - 1}}$$

The non-homogeneous nature of Cl^−^ adsorption on Fe should therefore also induce non-homogeneous and irregular Fe cation injection from substrate. Since the passivity of stainless steels is controlled primarily by the selective dissolution of iron^41^, we believe that the chloride-induced non-uniform cation injection of iron would obviously yield a passive film with an irregular and undulating Me/BL interface, as illustrated in Fig. 7b, wherein a large amount of chloride adsorption yields a faster growth of film, in contrast, chloride-free or little chloride leads to a slower growth. As a result, the concave sites, derived from the chloride attack, and the convex sites, with no chloride or little chloride, are formed.

As the oxide film thickens progressively, whether or not the concave sites are more aggressively attacked (yielding a faster film growth) or the convex sites more gently attacked (leading to a slower film growth), is decided by the facility of chloride ion permeation. That depends on two aspects: one is the electric field and the other is the intrinsic nature of the new oxide film. The electric field in the oxide varies during film growth^69,70^. The concave interfaces in the film are notably thicker and possess weaker electric field than the convex interface and hence pose greater resistance to chloride ion permeation. Correspondingly, parts of the metal substrate directly beneath the concave positions in the undulating film will experience less severe cation injection process, which retards film thickening. In contrast, cation injection process of the metal substrate beneath the convex positions is more pronounced, with the higher electric field speeding up the thickening of the passive film. Evidently, the effect of the electric field is against further amplifying the amplitude between the convex and concave locations.

Nevertheless, if the intrinsic nature of the thicker passive film located at some concave interfaces facilitates the chloride permeation, namely, it is less compact or exhibits more connected paths created along the NCs/amorphous interface, those concave sites can be expected to still keep a faster film growth, and vice versa for the convex interface. The cumulative impact of the occurrences ought to amplify some undulations (concave/convex locations), and the convex interfaces have the tendency to move closer and closer to the outer surface of the passive film, as illustrated in Fig. 7b.

Another mode of chloride attack, involving permeation through pathways along the NCs/amorphous zone interface, as mentioned earlier, is illustrated in Fig. 7c. Such a mode is only feasible when a connected path exists through the film. As shown in Fig. 7c, chloride ions will permeate the passive film laterally through the red tunnel and arrive at the matrix/ film interface. This accounts for the few select locations at the interface, where chloride ion accumulation was detected, with corresponding interface undulation.

According to the large amount of observation in this study, we do find that the role of interface roughening is quite non-uniform. In some locations, the roughening of interface is very considerable, as shown in the HAADF-STEM image of Supplementary Fig. 12. In the zoom-in image Supplementary Fig. 12(b), which is an enlarged image of the area marked with a rectangular in (a), the convex site at the matrix side (featuring with the well-defined lattice images) is getting close to the outer surface of the passive film, wherein, accordingly, the thickness of the passive film is quite thin. It is reasonable to propose that, with further roughening, the film would become thinner and thinner. Under the assistance of the stress, the film would be pulled apart and finally a breakdown would occur.

Pit nucleation, initiated at the surface of high purity metals or even of single crystals, is generally thought to be random and unpredictable. Our present experimental results and the analysis above indicate that convex sites where the nature of the passive film facilitates its amplification would be the preferential sites for film breakdown and pit nucleation.

Interestingly, it becomes obvious from our atomic-scale analysis of the chloride attack mechanism that the film breakdown sites are not really locations with high chloride ion concentration (concave sites) as widely believed but are actually the adjacent locations where the effect of chloride ions are relatively weak (convex sites). This idea, which is based on our experimental findings, introduces another dimension to understanding the mechanism of chloride attacking the passive film and implying the mechanism of chloride-induced passivity breakdown.

One plausible mechanism for the observed lattice expansion involves penetration and occupation of vacancies in the metal lattice by chloride (either chloride ions and/or Cl atoms), which both possess larger radii than Fe and Cr and as such can induce lattice expansion. Chloride can modify processes occurring at the interface when Cl adatoms (from Cl^−^ ion reduction) penetrate and occupy the residual Fe vacancies from the cation injection, thereby causing lattice expansion. Correspondingly, no lattice expansion or slight lattice expansion happens at the convex sites, while remarkable lattice expansion occurs at the concave sites (schematically illustrated in Supplementary Fig. 13). It is worth mentioning that the lattice behaviors based on our LADIA simulation reflect the atomic projection along the [100] direction. Such a projection makes the outmost layer at the matrix side actually correspond to the convex area with no or little lattice expansion, yielding the lattice expansion to be below the interface, rather than at the interface (Fig. 6b and Supplementary Fig. 13). Regarding the chloride presence below the interface at a depth where lattice expansion is measured, we did identify the chloride signal that is derived from the elemental maps, as shown in Supplementary Figure 14, although its intensity is much lower than that in the inner layer of the passive film.

All of our experimental results indicate clearly that chloride ions remarkably and unilaterally modify the interface zones via lattice expansion on the metal side and induced undulations at the interface and structural inhomogeneity on the film side. Such a series of events, by which chloride ions incorporate and attack the passive film, were neither envisaged nor considered probable in the available theories describing chloride-induced passivity breakdown.

We have shown that the passive films are mostly amorphous but with small amount of embedded NCs and that interfaces between NCs and the amorphous zone assume the features of grain boundaries providing ready paths for the chloride ion transport. We desire a passive film with full amorphous structure, so that tunnels for species diffusion and transport are not available. We could apply microalloying via an adding of certain element(s), which enhance the degree of amorphization of the passive film and in the meanwhile the properties of bulk steels remain unchanged. The possibility of this proposal is under consideration and there is no doubt that the newly reported mechanism will prompt scientists and engineers to reconsider the existing models and look for all the possible approaches to retard the passivity breakdown.

In summary, this study has provided new experimental insights on the well-known role of chloride ions in passivity breakdown, where the existing ideas are mostly based on theoretical models, with insufficient experimental evidence. Using spherical Cs-corrected TEM, in combination with computational modeling, we have compared the passive films formed in chloride-free and -containing electrolytes and directly observed atomic-scale accumulation of chloride ions at the metal/film interface, including the chloride-induced lattice expansion on the metal side, interfacial undulations, and structural alterations to the film. We find that the passive film is mostly amorphous, with some NCs. We directly visualize, from the in-plane and also the out-of-plane direction, the size, distribution, and crystallographic orientation of NCs and figure out the boundaries between NCs and the amorphous phase. Our experimental and computational results suggest that the interface between NCs and the amorphous zone assume a special kind of grain boundaries and thus provide ready paths for chloride ion transport. Our results show clearly that chloride ions actually permeate the outer and inner layers to attack the interface, rendering it indistinct and undulating. The weakest site which is believed to be the preferential site for a breakdown does not coincide, at atomic scale, with locations having high chloride ion concentration as widely believed but occurs at adjacent locations with subdued chloride ion influence.

## Methods

### Materials preparation

The FeCr_15_Ni_15_ (wt.%) single crystal alloy was grown by thermal-gradient directional solidification method. The orientation was determined by single-crystal X-ray diffractometry, and two low-index crystallographic orientations [001] and [110] were obtained. The (001) or (110) plane as exposure surface was potentiostatically passivated at a potential of 640 mV/SHE for 30 min in chloride-free and -containing electrolyte. The cross-sectional TEM specimen was prepared by the conventional method. Two passivated surfaces of two samples were bonded face-to-face and then thinned by grinding and ion-milling.

### HAADF-STEM imaging

HAADF-STEM images were recorded using Cs-corrected TEM (Titan Cubed 60–300 kV microscope (FEI) fitted with a high-brightness field-emission gun (X-FEG), double Cs corrector from CEOS, and a monochromator operating at 300 kV). The beam convergence is 25 mrad and thus yields a probe size of <0.1 nm.

### LADIA calculation method

The LADIA package was used to analyze the elastic strain state of the alloy underneath the passive thin film. This algorithm determines the displacement of actual column image positions versus the position of a reference lattice. From this information, we determined the local expansion/contraction of next-neighbor atom column distances in the alloy, i.e., the local lattice parameters in the <110> directions orthogonal to the <001> viewing direction and the <100> directions orthogonal to the <011> viewing direction.

### Data availability

The data that support the findings of this study are available from the corresponding author upon reasonable request.

## Electronic supplementary material

Supplementary Information

Peer Review Report
