# Supplementary material for: Unmasking chloride attack on the passive film of metals
Source: Nat Commun. 2018 Jul 2;9:2559. doi: 10.1038/s41467-018-04942-x (PMC6028649; doi:10.1038/s41467-018-04942-x)
Supplement: Supplementary file 1 — Supplementary Information [file 41467_2018_4942_MOESM1_ESM.pdf]

Unmasking chloride attack on the passive film of metals

Zhang et al.

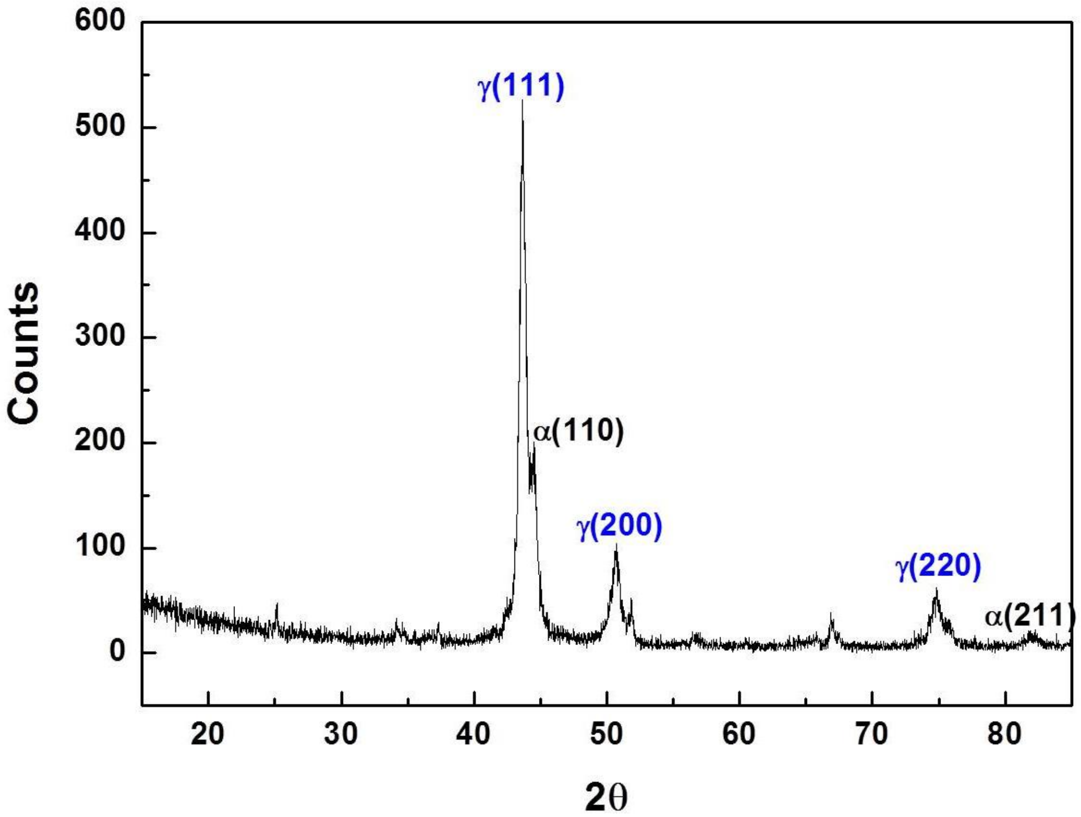

Supplementary Figure 1. The XRD analysis of the $FeCr_{15}Ni_{15}$ single crystal. The single crystal is mainly composed of the austenite phase.

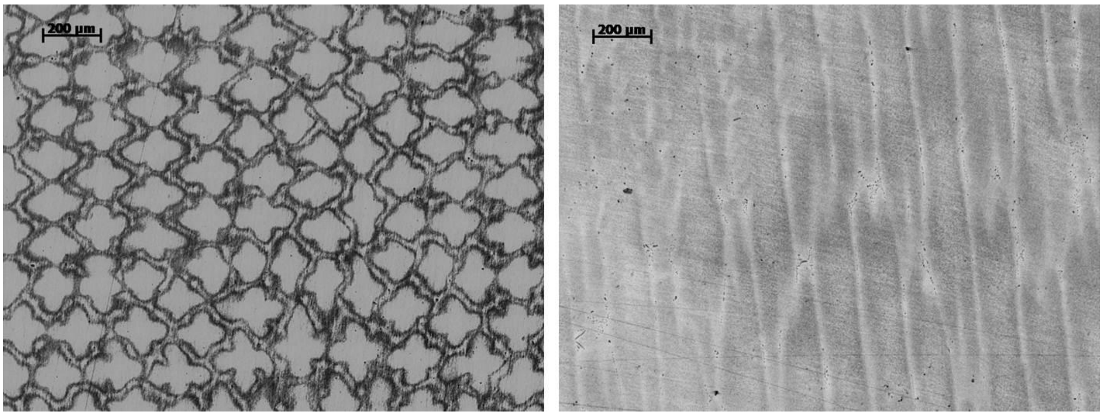

Supplementary Figure 2. Metallographic images of the $FeCr_{15}Ni_{15}$ single crystal perpendicular and parallel to the growth direction.

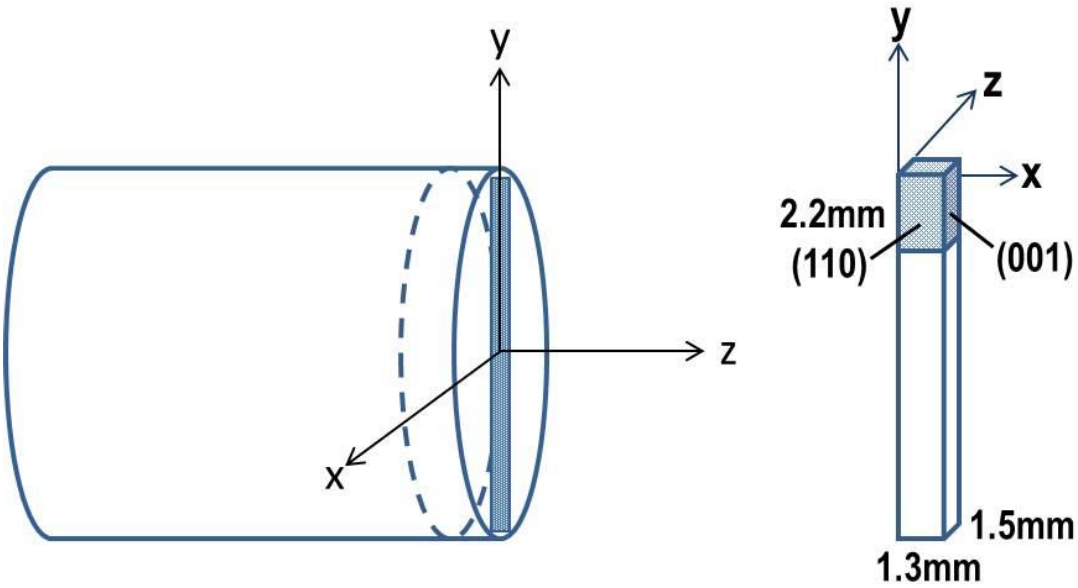

Supplementary Figure 3. Schematic of the procedure for cutting the single crystal alloy rod.

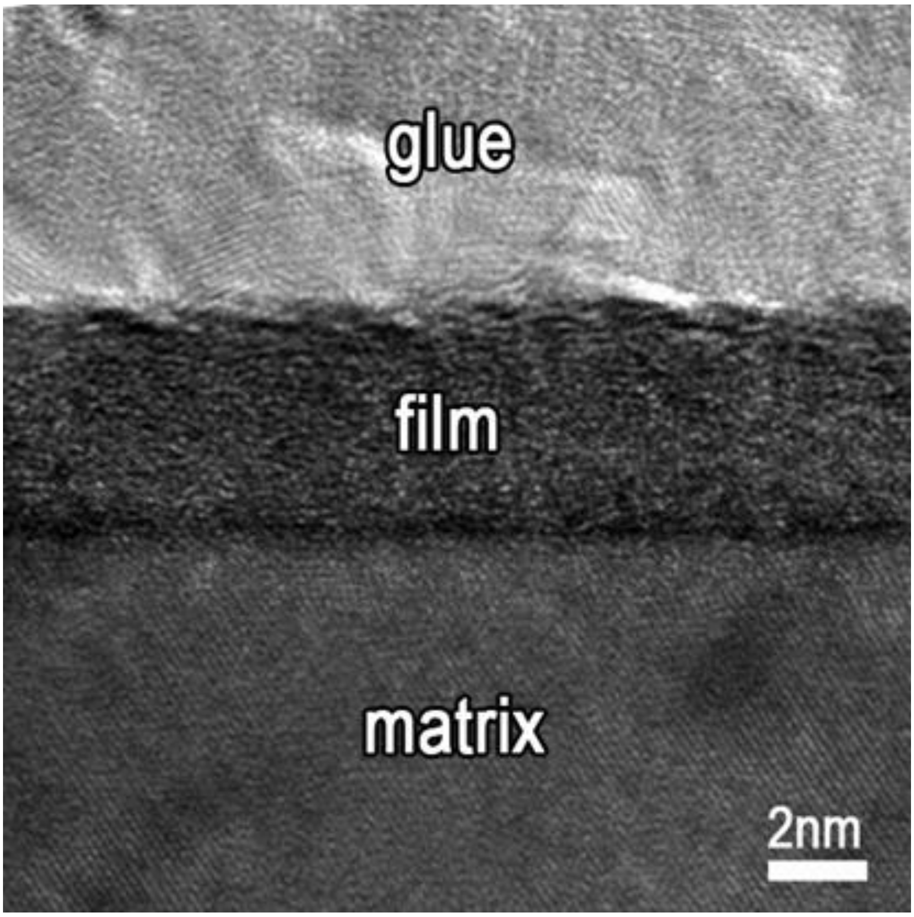

Supplementary Figure 4. A cross-sectional TEM image showing the passive film on the austenite $FeCr_{15}Ni_{15}$ matrix.

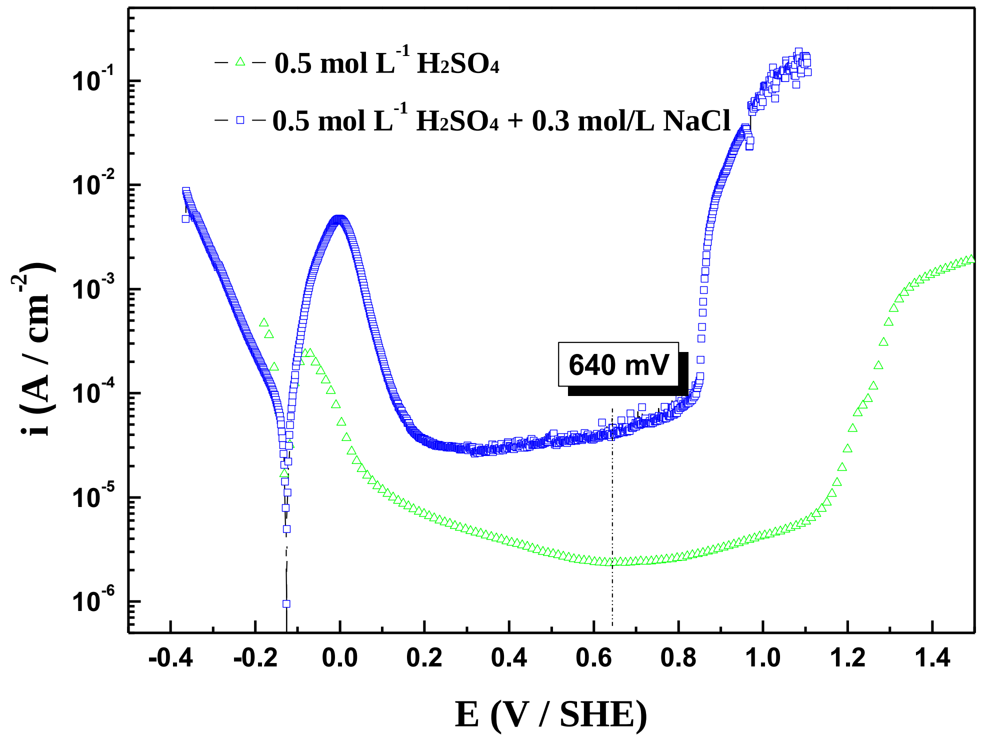

Supplementary Figure 5. Potentiodynamic polarization curve of $FeCr_{15}Ni_{15}$ in 0.5 mol L^{-1} H_2SO_4 and 0.5 mol L^{-1} H_2SO_4 + 0.3 mol L^{-1} NaCl electrolytes.

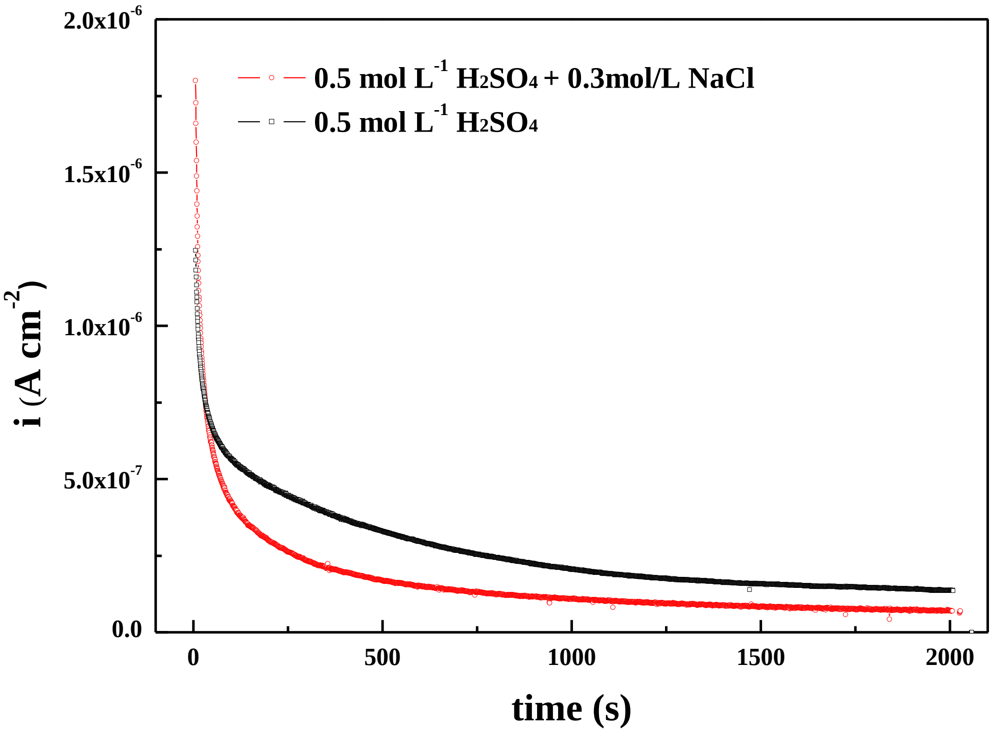

Supplementary Figure 6. Typical current/time curves for samples polarized at 640 mV / SHE in 0.5 mol L^{-1} H_2SO_4 and 0.5 mol L^{-1} H_2SO_4 + 0.3 mol L^{-1} NaCl.

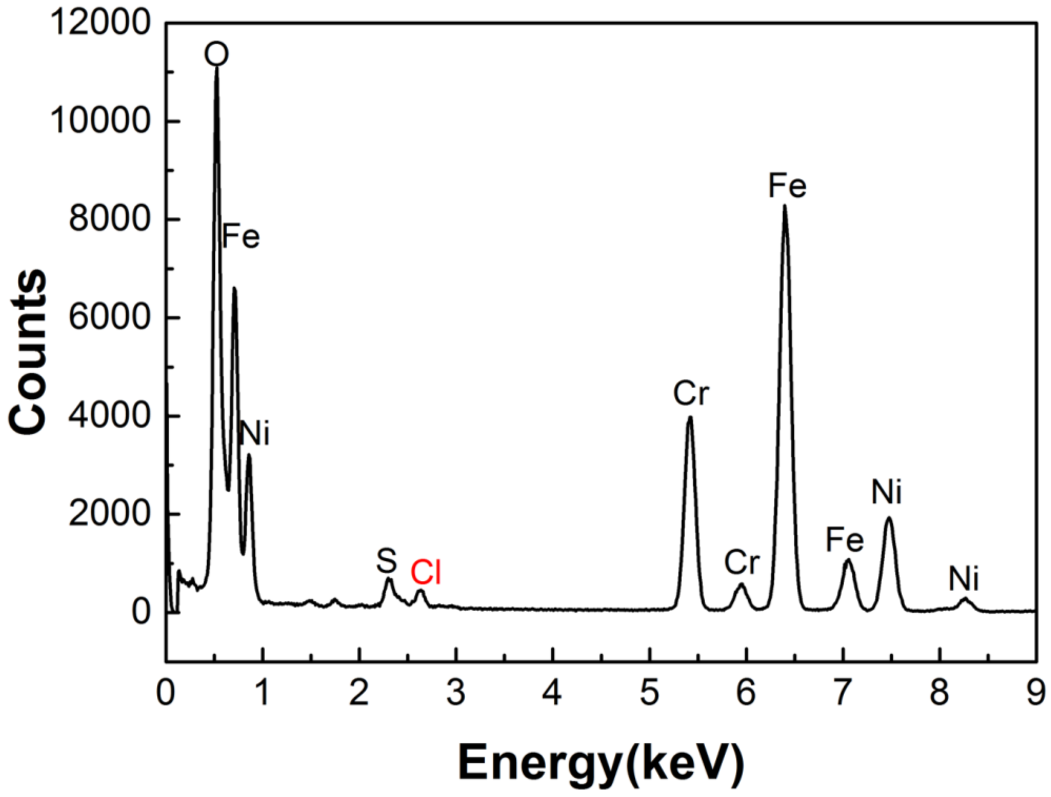

Supplementary Figure 7. EDS analysis on the inner layer of the passive film showing an evident element Cl peak.

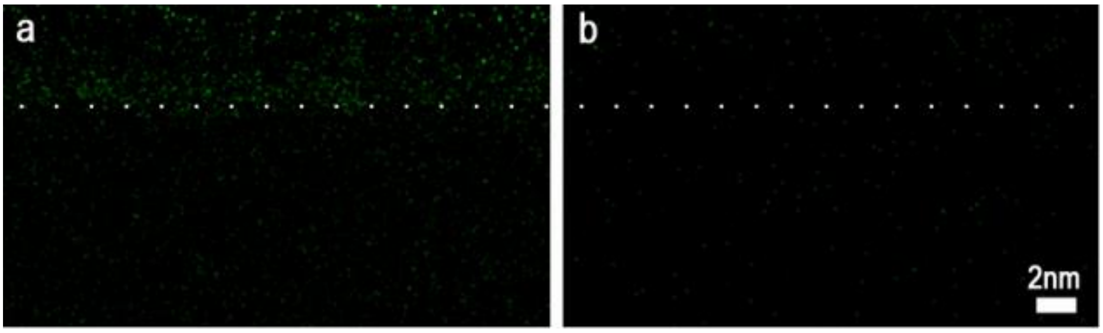

Supplementary Figure 8. EDS mapping of the passive film which was formed via initial passivation in H_2SO_4 electrolyte for 30 min and subsequent addition of NaCl, showing reduced prevalence of element Cl enrichment. (a) Element Cl is detected at locations manifesting the undulating interface. (b) Element Cl is not detected at the still distinct and unperturbed locations.

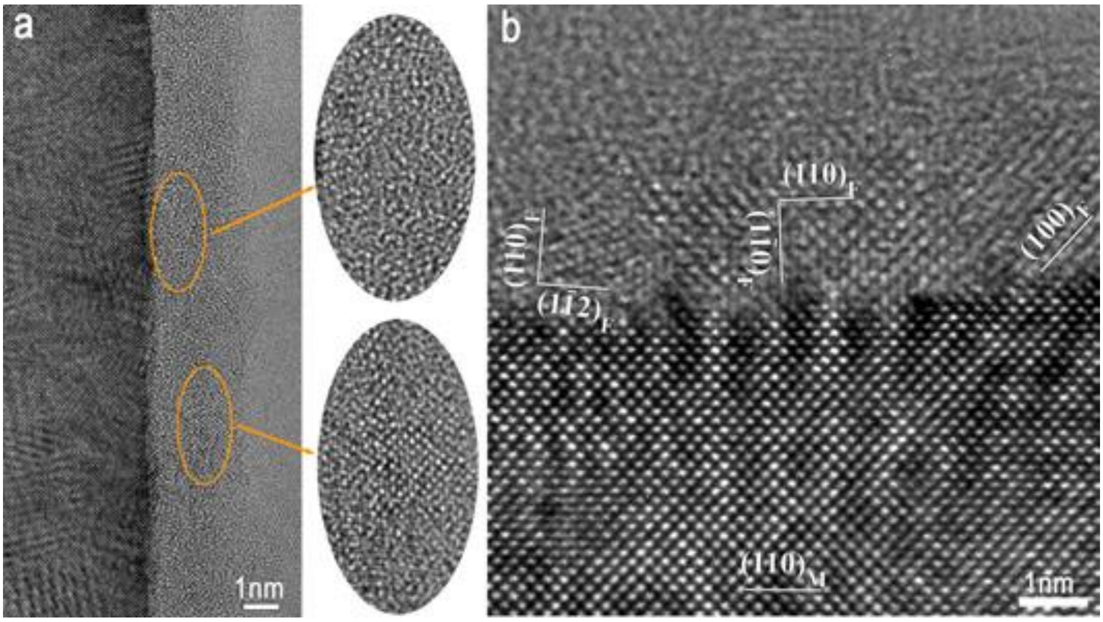

Supplementary Figure 9. (a) HRTEM images along the [001] axis of the austenitic matrix showing that the passive film is mainly amorphous with some nano-crystals. A series of HRTEM images obtained from variant orientations and locations indicate that the nano-crystals feature face-centered cubic structure (b) HRTEM images along [001] axis showing three fine crystals within the passive film.

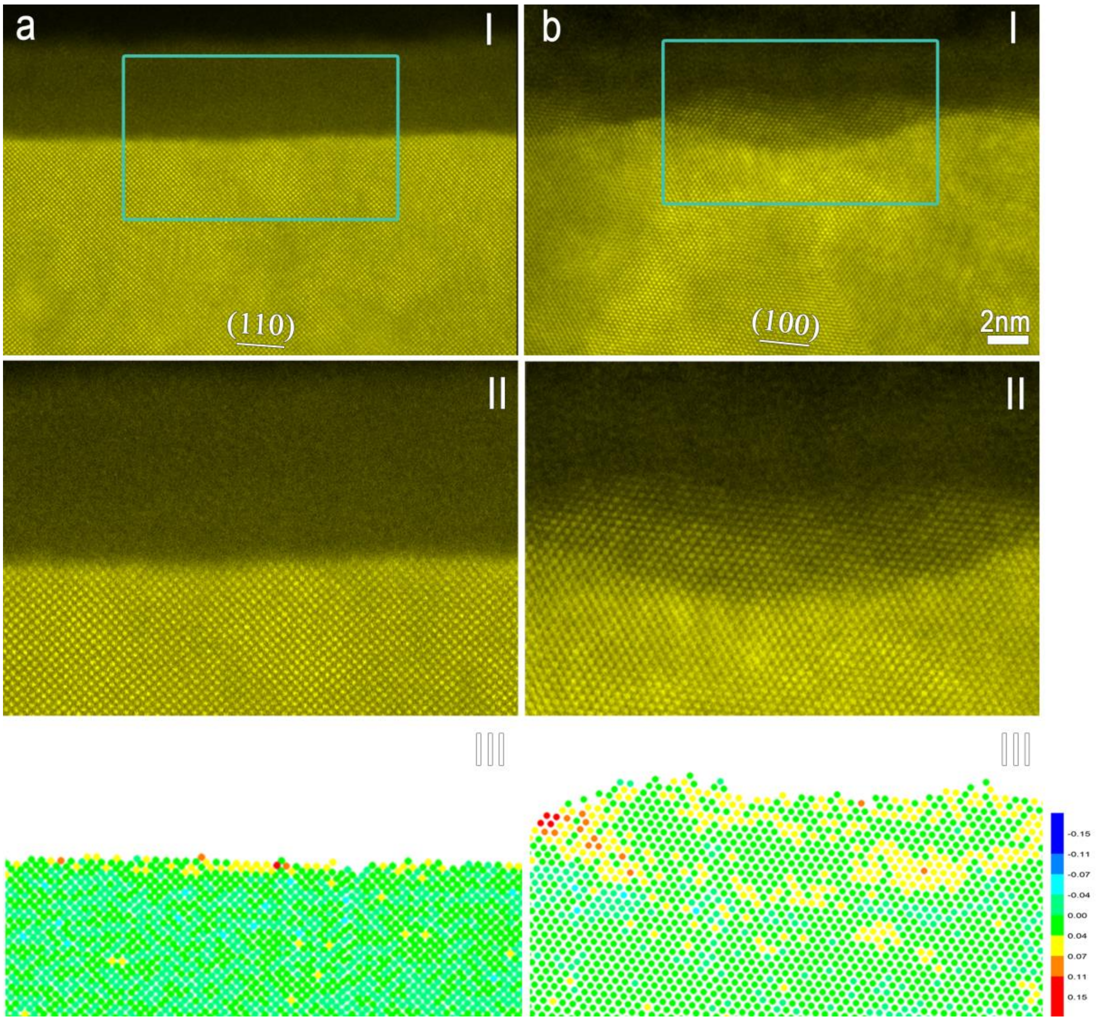

Supplementary Figure 10. LADIA simulation results showing the strain state in the matrix side near the metal/passive film interface. (a-I) High resolution HAADF-STEM image along the [001] axis showing a passive film formed in 0.5 mol L^{-1} H_2SO_4 electrolyte with straight interface. (a-II) Magnified (zoom-in) image of a-I. (a-III) LADIA simulation map based on a-II, which shows no evidence of lattice expansion and associated tension. (b-I) High resolution HAADF-STEM image along the [110] axis showing a passive film formed in 0.5 mol L^{-1} H_2SO_4+ 0.3 mol L^{-1} NaCl electrolyte, with corresponding undulating interface. (b-II) Zoom-in image of b-I. (b-III) LADIA simulating map based on b-II, revealing obvious lattice expansion, with associated induced tension. The colour bar on the right indicates the normal strain, where colours for positive values represent tensile strain and colours for negative values represent compressive strain.

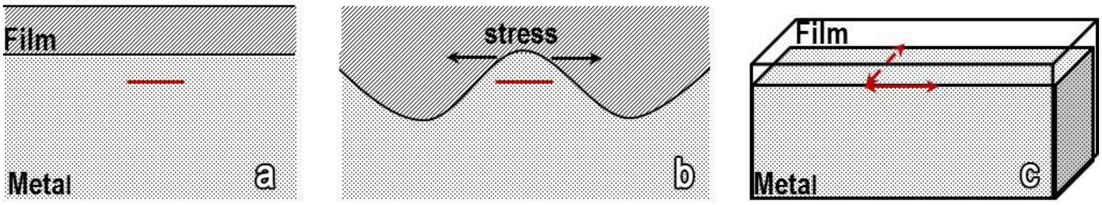

Supplementary Figure 11. Schematic maps illustrating the direction (red line) along which local expansion/contraction of next-neighbor atom column distances is modulated. (a) Straight interface formed in chloride-free electrolyte reveals no signs of lattice distortion on the matrix side; (b) Undulating interface formed in chloride-containing electrolyte shows clear evidence of strain-induced lattice expansion. Correspondingly, the tension at the interface is marked with a pair of arrows. (c) Three-dimensional schematic map illustrating the strain state along the viewing direction (a pair of dashed arrows) is equivalent to that as shown in (b).

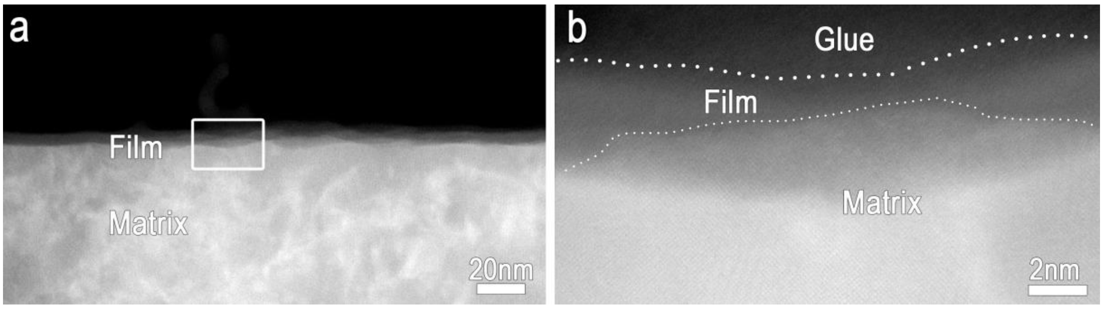

Supplementary Figure 12. Chloride-induced large roughening at the metal/passive film interface. Image in (b) is an enlargement of the area marked with a rectangular in (a).

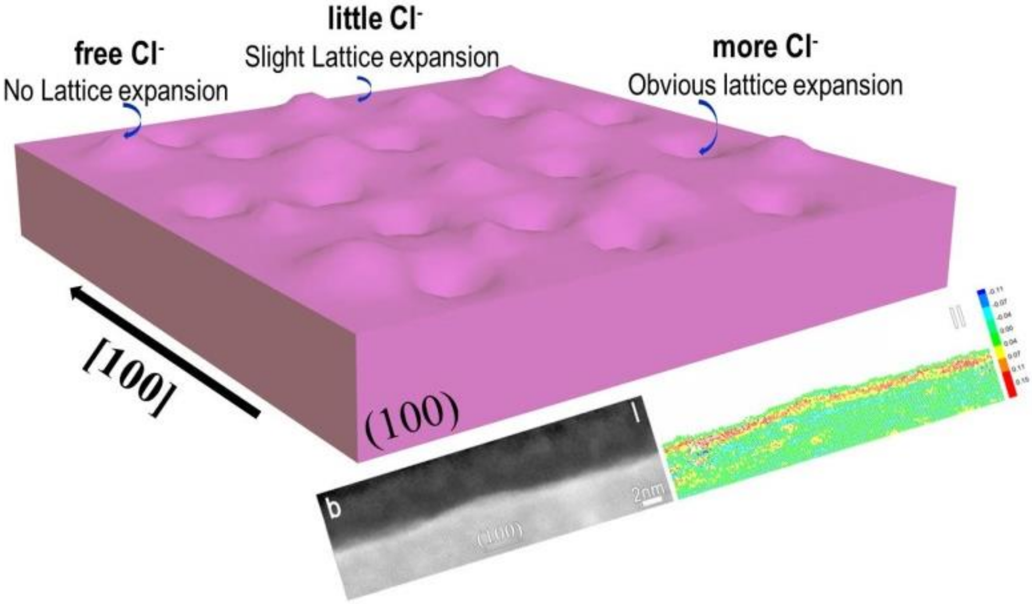

Supplementary Figure 13. Schematic map illustrating why, in the LADIA simulation image, the lattice expansion seems to be below the interface, rather than at the interface.

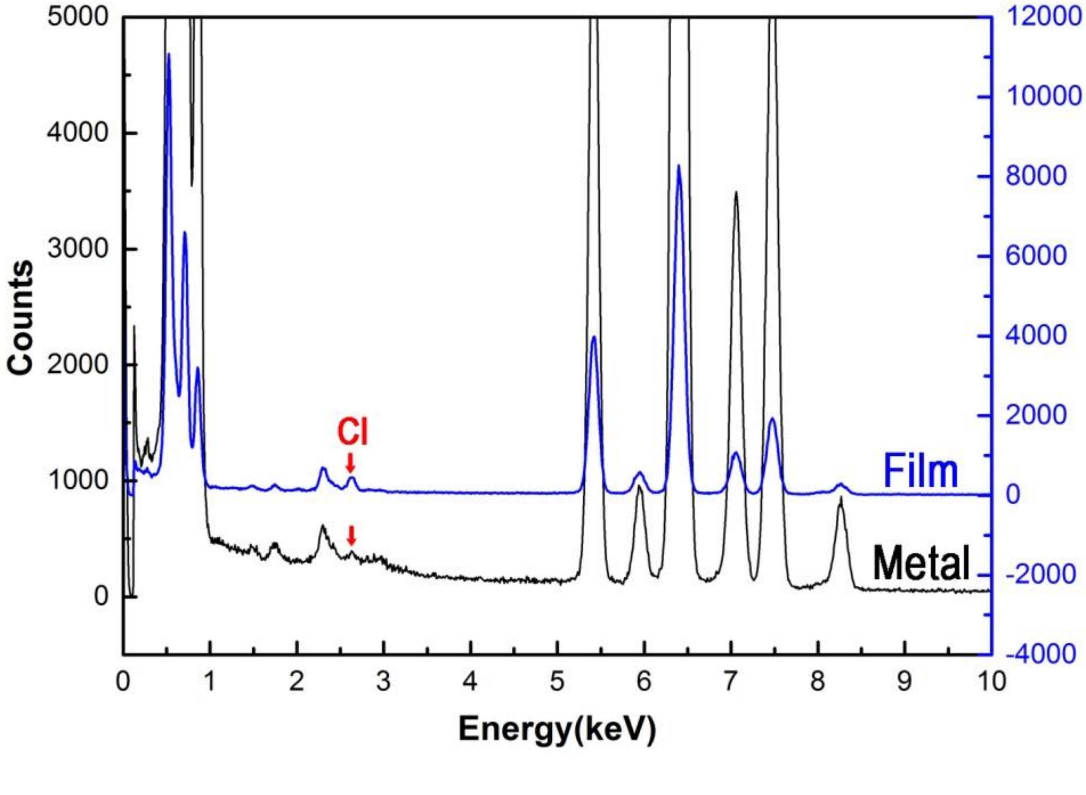

Supplementary Figure 14. EDS composition analysis in the passive film and below the interface at a depth where lattice expansion is measured. It is seen that the Cl peak, detected below the interface at a depth where lattice expansion is measured, is visible, although its intensity is lower than that in the inner layer of the passive film.

Supplementary Note 1

Single crystal alloy preparation. The $FeCr_{15}Ni_{15}$ (wt. %) single crystal alloy was used as matrix on which the passive film was formed for the study. The thermal gradient directional solidification method was used to grow the single crystal alloy. The single crystal can be treated as an austenite single phase alloy with trace amounts of other phase (Fig. S1). Metallographic images of representative morphologies of the directional solidification growing single crystal are given in Figure S2.

Supplementary Note 2

Orientation determination of the $FeCr_{15}Ni_{15}$ single crystal. The passive film has an amorphous structural character with some short-range ordering. Therefore, when observing the film/matrix interface by HRTEM, the matrix would be tilted to a zone axis. It is noteworthy that the passive film is ultrathin (3~5 nm) and thus tilting the matrix to a large angle would cause the matrix and the film to overlap, which will invalidate any observed interface structure. So, it is crucial that the direction of observation in TEM is just along a zone axis without any tilting, which normally necessitates cutting of the specimen along a specific crystallographic plane. The orientation was determined by single-crystal X-ray diffractometery. The single crystal rod was fixed on a clamper device which can tilt towards x, y and z direction spatially. We successfully obtained two low-index crystallographic orientations [001] and [110]. Then, the rod was cut into1.3 ×2.2 ×1.5 mm cuboid by means of a linear precision saw, with two adjacent orthogonal surfaces being (001) and (110) crystallographic planes, as shown schematically in Figure S3. During grinding of the cuboid specimens with varied grit silicon carbide papers, we tried our best to keep the surface level in order to avoid the artificial deviations from the crystallographic plane.

The introduction of single crystal enabled us to obtain a distinct metal/passive film interface and better characterize the structure of the interface region; on the other hand, single crystal is free of any inclusions and grain boundaries yielding a passive film high-quality with a continuous coverage on the alloy matrix, effectively avoiding the “the weakest sites breaking down the soonest” which makes figuring out the intrinsic mechanism complex.

Supplementary Note 3

Passive film formation. The ground cuboid specimens were polished electrochemically in $HClO_4$ (10 vol. %) + ethyl alcohol (90 vol. %) at 6~10V voltage,

to remove the deformed layer and obtain the smooth surface. Then the specimens with (001) or (110) plane as exposure surface were sealed with thread sealing tape and olefin resin to be the electrodes for subsequent passivation treatments.

AUTOLAB PGSTAT302N electrochemical workstation and a traditional three-electrode system were used in electrochemical experiments. The working electrode was the $FeCr_{15}Ni_{15}$ alloy, Pt counter electrode and $Hg/HgSO_4$ (saturated with K_2SO_4) reference electrode. All the potentials are described with reference to the standard hydrogen electrode (SHE). Potentiodynamic polarization measurements were performed firstly (shown in Fig. S5), with scan rate of 0.33 mV/s, in aerated 0.5 mol L^{-1} H_2SO_4 and 0.5 mol L^{-1} H_2SO_4 + 0.3 mol L^{-1} NaCl electrolytes respectively, to determine a suitable passivation potential (640 mV / SHE was selected to be the passive film formation potential) in the passive region for the potentiostatic passivation process. The specimen was depolarized at -1.2 V/SCE for 30s before potentiostatic passivating, which avoided the native oxide formation in air. Then the potential was stepped from the corrosion potential to 640 mV/SHE and maintained for 30 min at this potential, during which the i-t curves were recorded.

To track the effect of chloride ions on the passive film breakdown, distinct films were formed under three conditions: (1) passivated in 0.5 mol L^{-1} H_2SO_4 electrolyte at 640 mV/SHE for 30 min, (2) passivated in 0.5 mol L^{-1} H_2SO_4 + 0.3 mol L^{-1} NaCl electrolyte at 640 mV / SHE for 30 min, and (3) passivated in 0.5 mol L^{-1} H_2SO_4 electrolyte for 30 min with subsequent addition of NaCl into the H_2SO_4 electrolyte. The concentration of the subsequently added NaCl was calculated in such a way as to guarantee that the final concentration was commensurate with 0.3 mol L^{-1}.

It is noteworthy that, even the specimen is potentiostatically polarized in a chloride-containing electrolyte within the passive region, it is still in risk of breakdown of passive film and a subsequent repassivation process, which is usually reflected by the current transients in the i-t curve. The frequency of the transients represents the number of the breakdown-repassivation events.

In our study, we avoid an observation after the film had been broken down, this is because once a breakdown occurs, the metal would immediately exposure to the solution and the chloride ions might directly attack the metal. In such a case, it is impossible to identify the history of chloride-attacking upon the passive film. In other words, an observation after breakdown may make us miss the primary information on what really happened in terms of how and where chloride attacks the film leading to

the breakdown. Based upon the consideration above-mentioned, in our study only those specimens whose i-t curves had no any current transient, e.g. no any breakdown event occurred, were made into cross-sectional TEM specimens for further observation. This ensures the specific origin of chloride detected within the passive film.

Supplementary Note 4

TEM specimen preparation and technology. The cross-sectional TEM specimen was prepared by the conventional method. Two passivated surfaces of two samples were bonded face-to-face and then thinned by grinding, and ion-milling. The HRTEM and HAADF-STEM images were obtained by aberration-corrected transmission electron microscopy (Titan Cubed 60-300kV microscope (FEI) fitted with a high-brightness field-emission gun (X-FEG), double Cs corrector from CEOS, and a monochromator operating at 300kV).

Due to the extremely thin passive film with a thickness of only a few nanometers, ensuring the film free of damage during sample preparation and TEM observation is of critically important. After the cuboid (1.3 ×2.2 ×1.5 mm) specimens were polished electrochemically, the surfaces were strictly free of touch in the subsequent sealing, passivating, rinsing, sealing-tape removing steps. During bonding the two passivated surfaces of two samples face-to-face, slip between the two surfaces was avoided in fixation. After the cross-sectional specimen was thinned by grinding, we gave up the dimpling step and directly performed the ion-milling. By doing so, mechanical damage would be most possibly avoided.

The passive film is indeed less resistant to beam irradiation. During the first stage of TEM operation, optical alignment and aberration adjustment were performed at the location far away the target area. Generally, the most serious damage would be at the EDS-mapping experiment during which a large number of scanning points are necessary for ensuring the high resolution in composition distribution, which would yield long time beam irradiation to the film. In our experiment, we use the advanced Super-X EDS system with four detectors for the mapping analysis, which extremely shortens the experimental span and thus effectively avoids the beam damage to the film.

Supplementary Methods

Computational method. First principles calculations were performed to simulate the

diffusion of Cl^- anions within the crystalline Fe_3O_4(c-Fe_3O_4), amorphous Fe_3O_4 (a-Fe_3O_4) and the interface of c-Fe_3O_4/a-Fe_3O_4. It is noteworthy that the spinel Fe_3O_4 was treated as the simplified model of the passive film. The diffusivity of Cl^- anions in the c-Fe_3O_4, a-Fe_3O_4 and their interface regions can be approximated through the energy barrier overcome by Cl^- ion diffusing from one O vacancy to a neighboring one. Accordingly, a $1 \times 1 \times 3$ supercell was constructed for the interface between the crystalline and amorphous Fe_3O_4. Based on the interface model proposed by Monkhorst-Pack [1], we chose several O vacancy pairs in different positions and performed the calculations. In the amorphous and the interface zone, two pairs of O vacancies were chosen, while only one pair was chosen in the crystalline zone since all the O vacancy pairs in this zone are identical.

One Cl^- ion was inserted in one oxygen vacancy and the structure was relaxed. The same handle was repeated on the counterpart oxygen vacancy. When the two minima were found, we chose five points along the diffusion path and did the constrained minimization for each point. The one with the highest energy was chosen as the saddle point and the difference between this point and the lowest minimum was the diffusion barrier.

The calculations presented in this work are based on the density functional theory (DFT) as implemented in the Vienna ab-initio simulation package (VASP) [2]. The generalized gradient approximation (GGA) [3] exchange-correlation energy functional was used with projector augmented wave (PAW) method [4]. The GGA+U calculations were performed with the effective parameter of interaction between electrons U_{eff} = 3.61 eV according to the *Dudarev's* approach [5]. The plane-wave cut-off energy was chosen as 500 eV. The optimized lattice constants of the Fe_3O_4 bulk structure (8.48 Å), are consistent with previous experimental results (8.396 Å) [6]. The ionic relaxation was considered as convergence when the force on every atom is less than 0.05 eV $Å^{-1}$.

LADIA calculation method. The LADIA package [7, 8] was used to analyze the elastic strain state of the alloy underneath the passive thin film. This algorithm determines the displacement of actual column image positions versus the position of a reference lattice. From this information, we determined the local expansion/contraction of next-neighbor atom column distances in the alloy, i.e. the local lattice parameters in the <110> directions orthogonal to the <001> viewing direction and the <100> directions orthogonal to the <011> viewing direction.

Supplementary References

[1] Monkhorst H. J. & Pack J. D. Special points for Brillouin-zone integrations. *Phys. Rev. B.* **13,** 5188-5192 (1976).

[2] Kresse G. & Furthmüller J. Efficient iterative schemes for ab initio total-energy calculations using a plane-wave basis set. *Phys. Rev. B.* **54,** 11169-11186 (1996).

[3] Perdew J. P., Chevary J. A., Vosko S. H., Jackson K. A., Pederson M. R. & Singh D. J., Fiolhais C. Atoms, molecules, solids, and surfaces: Applications of the generalized gradient approximation for exchange and correlation. *Phys. Rev. B.* **46,** 6671-6687 (1992).

[4] Kresse G. & Joubert D. From ultrasoft pseudopotentials to the projector augmented-wave method. *Phys. Rev. B.* **59,** 1758-1775 (1999).

[5] Dudarev S. L., Botton G. A., Savrasov S. Y., Humphreys C. J. & Sutton A. P. Electron-energy-loss spectra and the structural stability of nickel oxide: An LSDA+U study. *Phys. Rev. B.* **57,** 1505-1509 (1998).

[6] Okudera H., Kihara K. & Matsumoto T. Temperature dependence of structure parameters in natural magnetite: single crystal X-ray studies from 126 to 773 K. *Acta Crystallogr. B: Structural Science* **52,** 450-457 (1996).

[7] Du K. & Rau Y. Lattice distortion analysis directly from high resolution transmission electron microscopy images - the LADIA program package. *J. Mater. Sci. Technol.* **18,** 135-138 (2002).

[8] Du K. & Phillipp F. On the accuracy of lattice-distortion analysis directly from high-resolution transmission electron micrographs. *Journal of microscopy* **221,** 63-71 (2006).
