# Supplementary material for: Unmasking chloride attack on the passive film of metals
Source: Nat Commun. 2018 Jul 2;9:2559. doi: 10.1038/s41467-018-04942-x (PMC6028649; doi:10.1038/s41467-018-04942-x)
Supplement: Supplementary file 2 — Peer Review Report [file 41467_2018_4942_MOESM2_ESM.pdf]

Reviewers' comments:

Reviewer #1 (Remarks to the Author):

The paper by Zhang et al presents high resolution TEM imaging of the interface between the passive film and metal matric on FeCrNi alloy. The experiments are challenging and the data appear robust, however I do not believe it is suitable for publication in Nature Communications for the following reasons:
i) it does not add significantly to our knowledge of Cl- effects. Incorporation of Cl- during passivation has been shown numerous times using XPS and SIMS analysis – whereas Cl- is not incorporated into the film if added 'post-passivation'. The authors have not fairly reviewed the literature on this point [note different behaviour has been observed for Al – the references to McCafferty's work should not be used for a discussion on stainless steel]
ii) the nano-crystalline nature is well-established, not just from STM (surface studies as referenced) but from TEM data from the 1960s – albeit in those studies the films were removed from the substrates (none of these references have been included - see J. Kruger et al). The discussion around 'amorphous = good' is not well founded in experiment - or a natural consequence of any data presented (pure Cr is much more resistant yet has a crystalline passive film). Suggestions for remediation of the behaviour by adding film modifiers is not explored in any detail.
iii) One might expect a roughened interface simply as a result of the much greater critical current for passivation observed in the polarization curve – indeed there is not discussion of the electrochemistry. Other related questions arise around the nature of the passive current itself and of current transients that have observed in the passive region using micro-electrochemical techniques (ie if the film is always in a transient state how do transport models play a role?)
iv) the arguments for eliciting a stress related film breakdown model are weak- and not well described.

Minor considerations include:
i) There is no discussion of possible film damage during sample preparation – or beam induced effects?
ii) The passivation potentials chosen were very high -why did the authors choose this value?

Reviewer #2 (Remarks to the Author):

This is a well-researched project that offers clear evidence for the role chloride plays in the initiation of localized corrosion processes on Fe-Ni-Cr alloys. I recommend the paper be published once the following issues have been addressed.
• Since it was conducted on single crystals, the study, while directly addressing how chloride may permeate a passive oxide layer, obviously avoids dealing with the many more likely micro/macro scale defects at which localized corrosion is most likely to occur. This should be made clear in the manuscript.
• While it is the chloride which reaches the alloy/oxide interface that damages the interface leading to the features eventually causing breakdown locations, one would expect to see a gradient of chloride ions throughout the oxide. Is there any evidence that this is the case?
• The authors state that only specimens which did not exhibit transients were examined. This suggests they are ignoring a primary pathway by which breakdown occurs. The frequency and importance of such transients should be discussed.
• The evidence for, and rationale of why, the alloy/oxide adopts an uneven structure are persuasive. However, the arguments offered to explain why these undulations (concave/convex locations) are amplified until breakdown occurred, as schematically illustrated in Figure 7(b), are not totally convincing. Since the interface is more aggressively attacked at locations which initially become concave, leading to a thicker oxide coverage, one might expect these locations to

eventually corrode more slowly. As the oxide thickened and the locations of SROs in this new oxide occur, would not the interfacial attack shift to the more convex locations leading to a general surface roughening rather than the enhancement of the undulations? On pages 8 to 10 the authors seem to argue both for and against such a roughening. This discussion needs to be clarified.
• The transfer of metal ions across the alloy/oxide interface is described as dissolution even when the oxide is well-developed. This does not seem an appropriate description at this interface. The term “cation injection” might be better.
A number of more minor issues should be addressed.
• Many of the paragraphs are up to, and even over, 1 page long, which makes the manuscript tedious to read in places.
• Presumably the oxides were grown in deaerated solutions?
• In the legend to Figure 3, it should be stated that the yellow line indicates the alloy/oxide interface. In Figure 3(c) it is not clear that this line faithfully tracks the interface. What criterion was used to define where this line should be?
• On page 6, it is stated that the interfaces between the SRO structures and the amorphous zone assume the features of grain boundaries. The authors should be more specific about the features they allude to.
• The significance of the colour bar in Figures 6 and S10 needs to be specified.
Supplementary Material
• It is stated that the passive film is ~ 3nm thick. This seems at odds with the TEM image in Figure S4 in which the film looks significantly thicker.
• “The specimen was depolarized at -1.2V for 30s before potentiostatic passivation which avoided the native oxide formation in air”. While this is a standard treatment, there is evidence that such native oxides are not removed, only rendered highly defective. The authors should comment on this and whether it would affect their study.

Reviewer #3 (Remarks to the Author):

The focus of this work appears to be on the corrosion mechanism of FeCr15Ni15 alloy. The research topic is of great importance itself, while certainly of involving complicated factors that often are hard to be sorted out. Checking into the details of the present work, it is apparent that the authors have captured some details regarding the structure and chemistry of the passivation film. Taken from there, the authors have attempted to interpret the accelerated corrosion with the presence of chlorine, and from that point, the manuscript appears to be driven toward divergence, with apparent mishandling of fundamentals on corrosion science. The general conclusion regarding the chlorine transport in the amorphous region between the crystallized domains would be different from that of a whole amorphous phase is apparently a wrong one.

Overall, the TEM observation of passivation film structure and chemistry appears to be solid, while the interpretation are definitely full of assertive assumptions, and tangled in concept. Apparently the present work partially answers the question as what it is (the product), but hardly, and correctly, answers the questions as how and why.

Reviewer #4 (Remarks to the Author):

The key experimental claims of the paper , concerning the accumulation of chloride ions at the metal/film interface , the distortion of the interface and the strain in and local structure of the amorphous oxide are all very significant and worthy of publication.The experimental work is of high quality and makes a convincing story .

However the theoretical analysis does not match the quality of the experimental work. There are

major conclusion drawn from the literature (first paragraph) that are boldly asserted and not supported by any analysis. The section on chloride ion attack mechanism is really just a restatement of the observed experimental points without any substantive theoretical enhancement or analysis . Similarly in the interface strain distribution modelling the authors make bold claims without analytical justification - e.g chloride ions impart tension that can lead to film breakdown. The later statement is very important and may be true but is not justified.

The problems with the theoretical analysis is confounded by two other factors - the authors do not in any substantive way relate their observations but to the detail of any of the historically postulate mechanism and the reference to both theoretical and experimental developments are in the main part prior to the year 2000.
one minor point in the introduction the authors claim that no technique has looked at the evolution of the passive film etc. This is strictly true but a number of authors have carried out similar studies where they look at passive films at set times in a similar way to the authors.
Having made this comments i do believe that the experimental advances are significant and make the paper worthy of publication if the theoretical could be addressed. I would suggest significantly deepening the analysis or stripping it back and presenting a solely experimental paper.

Response to the referees' comments:

Reply to referee #1:

We appreciate the general recognition by the referee that "The paper by Zhang et al presents high resolution TEM imaging of the interface between the passive film and metal matric on FeCrNi alloy. The experiments are challenging and the data appear robust". However, the referee also raises some professional questions and comments which are summarized into four major aspects. We fully understand the referee's concerns, and here we address all the questions and discuss all the comments one-by-one in the following.

Questions/comments (1): it does not add significantly to our knowledge of Cl^- effects. Incorporation of Cl^- during passivation has been shown numerous times using XPS and SIMS analysis – whereas Cl^- is not incorporated into the film if added 'post-passivation'. The authors have not fairly reviewed the literature on this point [note different behaviour has been observed for Al – the references to McCafferty's work should not be used for a discussion on stainless steel].

Response to Questions/comments (1):

We appreciate the concerns raised by the referee. The significant contribution of the present study to our knowledge of Cl^- effects is that we have directly captured the whole spectrum, on the basis of real space cross-sectional imaging, of the chloride ion accumulation at the metal/film interface, as well as modifications at the interface zones via lattice expansion on the metal side, undulations at the interface and structural inhomogeneity on the film side. A series of these events resulting from the "incorporation of chloride ions" were neither envisaged nor considered probable in the available theories describing chloride-induced passivity breakdown.

Indeed, incorporation of Cl^- during passivation has been shown numerous times using XPS and SIMS analysis. Nonetheless, as stated in the 'Introduction' section in our manuscript, owing to the resolution limitation, XPS, AES and SIMS techniques are only able to provide an average value over the relatively larger area (typically from μm^2 to mm^2 scale). It is very difficult and challenging to guarantee the precision and accuracy of observed locations and concentrations of a very small amount of chloride in an extremely thin passive film with a thickness of only a few nanometers. In contrast, by means of aberration-corrected

high-resolution transmission electron microscopy (TEM), we have simultaneously investigated the film and metal matrix as well as their interface via cross-sectioning in the real space. We find the chloride accumulation within the inner layer of the passive film and the associated fluctuations at the matrix/passive film interface. We provide direct evidence on the location of chloride and the resultant phenomena which might be already there but undetectable in the past.

Much of evidence presented in the literature on the incorporation of Cl^- in the passive oxide film on Fe, Ni, Fe-Cr alloys and stainless steels can be mainly classified into two groups: one is chloride incorporation[1-10], and the other is chloride absence in the passive film [6,11-17]. In case of Cl^- incorporation, the chloride has been most often identified to be located or concentrated in the outer layer of the film[1-6].

The film formation procedure in our present study is identical to that in the literature, all of which include the potentiostatic polarization in chloride-free, chloride-containing electrolytes and passivation in chloride-free electrolyte with subsequent addition of chloride. This offers the same bias on which we can fairly compare our present observations by high-resolution TEM and previous identification by AES, XPS, SIMS, etc.

In some of the earlier studies, the specimens for film formation and the subsequent AES analysis were mechanically ground with an abrasive paper[6] or mechanically polished to 1um finish[4,5]. This would result in a surface with the roughness at micro-meter scale. Electropolishing[2,3] would also inevitably bring a nano-meter scale roughness. The Auger depth profiles therein, obtained over a large area (an example of 2×2 mm), were the average value over the relatively larger area. As a result, even if chloride ions are present in the inner layer of the passive film, the most pronounced peak of chloride would appear at the depth far from the metal/film interface, as we schematically illustrate appended below in Figure R1.

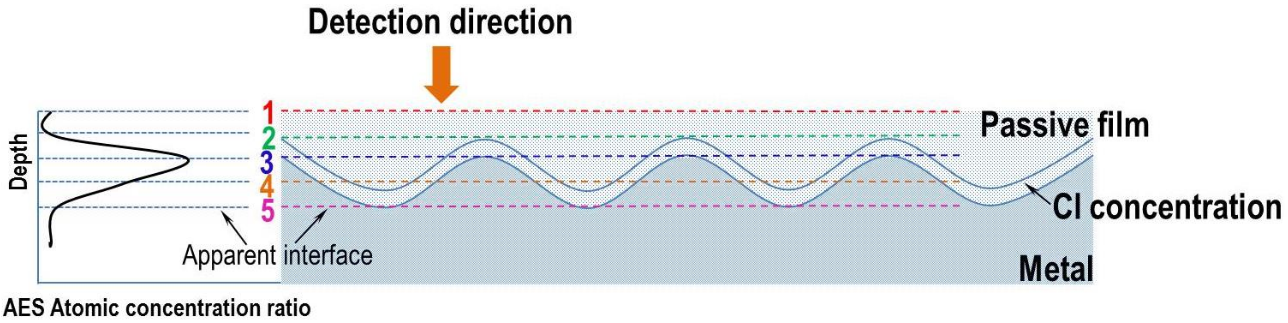

Fig. R1 Schematic illustrating the possible origin of error in the depth distribution of chloride using XPS and AES techniques.

In contrast, in our present study, the specimens were electrochemically polished, yielding a rather smooth surface; on the other hand, the cross-sectional TEM observations enable us to investigate the details across the metal/film interface at a high spatial and energy resolution, to visualize how the structure and composition develop from the metal to the film.

Another advantage of cross-sectional TEM observation is that we have opportunity to directly visualize the entire film no matter how thin the film is. We emphasize that, based on the advanced analytical tool with high spatial resolution, we provide direct evidence not only on the distribution of chloride in the film but also on the chloride-induced structural evolution at the interface, which might be already the case but undetectable in the past.

Regarding the pre-passivated specimens, indeed some investigators failed to find chloride in the passive films on pre-passivated specimens. We propose that one of the reasons might be the low concentration of the chloride incorporated into the passive films. It is predictable that the average concentration of chloride in the passive films on pre-passivated specimens must be much lower than that in the films which was formed at the presence of chloride in the electrolyte. Actually, in our present study of the pre-passivated specimens, we have observed a reduced prevalence of the undulating interface, which only manifests at some locations (as seen Fig. 3d in the text file). These exceptional locations are expected to be the terminal points for paths through which chloride ions permeate. Our Super-X EDS analysis indicates that the chloride is only detected at locations manifesting the undulating interfaces (typically like that in Fig. 2c) rather than at the segment of interface which remains distinct (as shown in Fig. S8). That is to say, chloride ions penetrate the film in a rather inhomogeneous manner. Such a local existence and low average concentration made them hardly detected by means of AES technique which has been widely used in this field.

According to the kind suggestion of the referee, we have supplemented a fuller set of references to previous relevant work, making the introduction and contextualization of the study rich of insight (see page 2, second paragraph, line 2-18). In the meanwhile, the references to McCafferty's work on Al (*Corros. Sci.* **37**, 481-492, (1995); *Corros. Sci.* **45**, 1421-1438, (2003).) are removed.

References in Response to Questions/comments (1):

1. D. F. Mitchell, *Appl. Surf. Sci.* **9**, 131 (1981).

2. B. MacDougall, D. Mitchell, G. Sproule, M. Graham, *J. Electrochem. Soc.* **130**, 543 (1983).

3. I. Olefjord, B. Brox, and U. Jelvestam, *J. Electrochem. Soc.* **132**, 2854 (1985).

4. A. R. Brooks, C. R. Clayton, K. Doss, and Y. C. Lu, *J. Electrochem. Soc.* **133**, 2459 (1986).

5. S. Mischler, A. Vogel, H. J. Mathieu, and D. Landolt, *Corros. Sci.* **32**, 925 (1991)

6. C. Hubschmid, D. Landolt, H.J. Mathieu, *Fresenius J. Anal. Chem.* **353**, 234 (1995).

7. O. Murphy, J.M. Bockris, T. Pou, L. Tongson, M. Monkowski, *J. Electrochem. Soc.* 130, 1792 (1983).

8. I. Olefjord and L. Wegrelius, *Corros. Sci.* **31**, 89 (1990).

9. P. Marcus, J.M. Herbelin, *Corros. Sci.* **34**, 1123 (1993).

10. S. Ningshen, U. K. Mudali, V. K. Mittal, and H. S. Khatak, *Corros. Sci.* **49**, 481 (2007).

11. W. Khalil, S. Haupt, and H. H. Strehblow, *Werkst. Korros.* **36**, 16 (1985).

12. R. Goetz, B. MacDougall, and M. J. Graham, *Electrochim. Acta* **31**, 1299 (1986).

13. V. Mitrovic-Scepanovic, B. MacDougall, and M. J. Graham, *Corros. Sci.* **27**, 239 (1987).

14. D. Landolt, S. Mischler, A. Vogel, and H. J. Mathieu, *Corros. Sci.* **31**, 431 (1990).
15. C. Hubschmid and D. Landolt, *J. Electrochem. Soc.* **140**, 1898 (1993).
16. C. O. A. Olsson and D. Landolt, *Electrochim. Acta* **48**, 1093 (2003).
17. P.M. Natishan, W.E. O'Grady, F.J. Martin, *J. Electrochem. Soc.* **158,** C7 (2011).

Questions/comments (2): the nano-crystalline nature is well-established, not just from STM (surface studies as referenced) but from TEM data from the 1960s – albeit in those studies the films were removed from the substrates (none of these references have been included - see J. Kruger et al). The discussion around 'amorphous = good' is not well founded in experiment - or a natural consequence of any data presented (pure Cr is much more resistant yet has a crystalline passive film). Suggestions for remediation of the behaviour by adding film modifiers is not explored in any detail.

Response to Questions/comments (2):

Indeed, several techniques have been applied in determining the structural information of the passive films. These include the real space imaging upon the outmost surface by STM technique[1-7], and reciprocal space identification by XRD analysis (by means of film isolation method)[8,9], electron diffraction in a TEM (also by means of film isolation method)[10-12]. In addition, extended X-ray absorption fine structure (EXAFS) analysis[13-21] was also applied to determine the structure of passive films.

In the original version of our manuscript, the reason that we emphasized the previous STM investigation and neglected the electron diffraction studies is that we wanted to compare the real space "direct imaging" between earlier STM and our present HRTEM.

Anyway, we appreciate the referee's reminder on these earlier experiments via TEM and we have added some earlier papers on TEM examination (page 7, line 21, and Refs. 83-85 in this revised manuscript) into the fuller set of references in this revised text.

Regarding the role of fully amorphization in chloride attack to passive films, we appreciate the concerns raised by the referee. We also believe that this is one of the central issues in this field. Our present study indicates that the film is composed of SRO (short range ordered structure) and amorphous phase. Our experimental and computational results suggest that the interface between SRO structures and the amorphous zone assume a special kind of grain boundaries and thus provide ready paths for chloride ion transport. This is also based on the fact that the chloride ions actually permeate the passive film heterogeneously.

Such a selective permeation arises from the inherently non-homogeneous microstructure of passive films and depends on the nature of and interconnection between the paths created along the SRO/amorphous zone interface. When a connected path traverses the entire thickness of the passive film, chloride ions tunneling through those paths could eventually reach the matrix/passive film interface. On the other hand, where connected paths are not available, or all the paths are abridged, the matrix/passive film interface would remain

unperturbed because chloride ions are unable to get through.

From the viewpoint of the role of grain boundaries in the films, our results are fundamentally consistent with the previous recognition that grain boundaries usually provide tunnels for species diffusion and transport yielding less resistant to Cl^- ion attack, although the grain boundaries therein were referred to the boundaries between crystalline oxide grains in the passive film since the analysis were almost based on the well-crystallized passive film on pure iron.

The role of grain boundaries in passivity breakdown and initiation of localized corrosion has been widely discussed. A number of models are available to describe the growth and breakdown of passive films. Almost all of the models involve the "ionic transport within the passive film", which is assumed to be highly dependent on the structure of film. The earlier models usually assumed the homogeneity of the films. Later, X-ray diffraction (XRD)[22,23] and scanning tunneling microscopy (STM)[1,24] studies clearly showed a nanocrystalline film on iron, which promoted the concerns that grain boundary might be potentially important for the ionic transportation within the passive film. A recent DFT calculation[25] further highlighted the role of grain-boundary in ionic transport within iron oxide by fact that growth kinetics models could be reconciled with experimental data only if passive film grain boundary effects were dominant. More recently, P. Marcus *et al* [26,27] proposed a model of passivity breakdown including the role of the inter-granular boundaries of the barrier oxide layer. They assumed that the grain-boundaries sites govern the potential drop and thus play the important role in the local thinning at film/metal interface; they also proposed that Cl^- can penetrate in the barrier layer via the inter-granular boundaries and migrate to the film/metal interface inducing film breakdown.

In a study of Fe-Cr-Ni alloys, Bertocci and Kruger considered that amorphous passive film was more protective than a crystalline one by the fact that an increase in Cr content in Fe-Ni alloys increases the tendency to form glassy oxide films and as well as the resistance to attack[28]. Based on that opinion, they evaluate the resistance to attack of the passive film formed on amorphous and crystallized Fe-Cr-Ni alloys using electrochemical noise technique and concluded that the latter has a much greater tendency to localized attack because of structural inhomogeneity of the passive film.

In the case of Cr, we agree with the referee that pure Cr is much more resistant yet has a crystalline passive film. However, we emphasize that the resistance difference between crystallized and amorphous film should be referred to the same substrate. Actually, Maurice *et al* examined the passive film formed on Cr (110) single-crystal surface and found the film is comprised of the larger ordered domains in the inner part surrounded by the non-periodic structural areas in the outer part[29]. They proposed the noncrystalline outer layer minimized the sites of passivity breakdown.

In summary, whether a grain boundary is referred to the boundaries between crystalline oxide grains or between SRO structures and the amorphous zone, so far many studies indicate that amorphization have greater tendency to resist localized attack. Thus, it is reasonable to propose that we desire a passive film with full amorphous structure (particularly for the outer layer of a film), so that tunnels for species diffusion and transport are not available. Regarding a technical solution to realize such a proposal, we suggest a microalloying via an adding of certain element(s) which enhance the degree of amorphization of the passive film and in the meanwhile the properties of bulk steels remain unchanged. Nevertheless, we fully understand that this need an extensive collaboration between researchers in several disciplines particularly in metallurgy, electrochemistry, and materials science. At the present stage, we expect that the new mechanism would stimulate relevant scientists and engineers to reconsider the existing models and look for all the possible approaches to address the problem of the passivity breakdown.

In the 3rd paragraph (written in red) at Page 7 of this revised version, we have briefly summarized the earlier studies on structural information on the nano-crystalline nature of the passive films, and the role of grain boundaries in passivity breakdown and initiation of localized corrosion.

References in Response to Questions/comments (2):

1.M. P. Ryan, R. C. Newman, G. E. Thompson, *J. Electrochem. Soc.* **142**, L177 (1995).

2. V. Maurice, W. Yang, P. Marcus, *J. Electrochem. Soc.* **141,** 3016 (1994).

3. M. P. Ryan, R. C. Newman, G. E. Thompson, *Philos. Mag.* **70,** 241 (1994).

4. M. P. Ryan, R. C. Newman, G. E. Thompson, *J. Electrochem. Soc.* **141,** L164 (1994).

5. V. Maurice, W. P. Yang, P. Marcus, *J. Electrochem. Soc.* **146**, 1182 (1996).

6. V. Maurice, W. P. Yang, P. Marcus, *J. Electrochem. Soc.* **145**, 909-920, (1998).

7. T. Massoud, V. Maurice, L. H. Klein, P. Marcus, *J. Electrochem. Soc.* **160**, C232-C238, (2013).

8. U. R. Evans, *J. Chem. Soc.* **127**, 1020 (1927).

9. W. H. J. Vernon, F. Wormwell, T. J. Nurse, J. Chem. Soc. 621 (1939).

10. J. E. O. Mayne, M. J. Pryor, J. *Chem. Soc.* **JUL,** 1831, (1949).

11. C. L. Foley, J. Kruger, C. J. Bechtoldt, *J. Electrochem. Soc.* **114,** 994 (1967).

12. C. L. McBee, J. Kruger, *Electrochim. Acta* **17,** 1337 (1972).

13. G. G. Long, J. Kruger, D. R. Black, M. Kuriyama, *J. Electroanal. Chem. Inter. Electrochem.* **150,** 603 (1983).

14. G. G. Long, J. Kruger, D. R. Black, M. Kuriyama, *J. Electrochem. Soc.* **130**, 240 (1983).

15. G. G. Long, J. Kruger, M. Kuriyama, D. R. Black, *J. Electrochem. Soc.* **130,** C314 (1983).

16. J. Kruger, G. G. Long, *J. Electrochem. Soc.* **133,** C302 (1986).

17. J. Kruger, *Inter. Mater. Rev.* **33,** 113 (1988).

18. J. Kruger, *Corros. Sci.* **29,** 149 (1989).

19. J. Kruger, G. G. Long, Z. Zhang, D. K. Tanaka, *Corros. Sci.* **31,** 111 (1990).

20. M. Kerkar, J. Robinson, A. J. Forty, *Faraday Discuss. Chem. Soc.* **89**, 31 (1990).

21. J. Robinson, F. C. Walsh, *Corros. Sci.* **35,** 791 (1993).
22. M. F. Toney, A. J. Davenport, L. J. Oblonsky, M. P. Ryan, C. M. Vitus, *Phys. Rev. Lett.* **79**, 4282 (1997).
23. A. J. Davenport, L. J. Oblonsky, M. P. Ryan, M. F. Toney, *J. Electrochem. Soc.* **147**, 2162 (2000).
24. E. E. Rees, M. P. Ryan, D. S. MacPhail, *Electrochem. Solid-State Lett.* **5**, B21, 2002.
25. S. C. Hendy, N. J. Laycock, M. P. Ryan. *J. Electrochem. Soc.* **152,** B271 (2005).
26. P. Marcus, V. Maurice, H. H. Strehblow. *Corros. Sci.* **50,** 2698 (2008).
27. A. Seyeux, V. Maurice, P. Marcus, *Electrochem. Solid State Lett.* **12,** C25 (2009).
28. U. Bertocci, J. Kruger, *Surf. Sci.* **101,** 608 (1980).
29. V. Maurice, W. P. Yang, P. Marcus, *J. Electrochem. Soc.* **141,** 3016 (1994).

Questions/comments (3): One might expect a roughened interface simply as a result of the much greater critical current for passivation observed in the polarization curve – indeed there is not discussion of the electrochemistry. Other related questions arise around the nature of the passive current itself and of current transients that have observed in the passive region using micro-electrochemical techniques (ie if the film is always in a transient state how do transport models play a role?)

Response to Questions/comments (3): We appreciate the concerns raised by the referee. From the viewpoint of the electrochemistry, the roughened interface can be understood to be a result of the much greater passive current (as shown in Fig. S5). Exactly, the greater passive current means much more dissolution during passivation in chloride-containing electrolytes. We believe that the chloride-induced non-uniform cation injection of iron would obviously yield a passive film with an irregular and undulating Me/BL interface, seen in the text of Page 9. Nevertheless, the interface roughening in the pre-passivated specimens is believed to result from the heterogeneous chloride permeation across the film to the metal/film interface. Indeed, discussions on electrochemistry have been included when addressing the formation process of the roughened interface.

As shown in Fig. S5, in the chloride-free electrolyte, a smooth polarization curve with a wide passive potential region indicates a relatively homogeneous dissolution of the substrate across the passive film. In contrast, in the chloride-containing electrolyte, some current transients appear in the passive range. The current transients (potentiodynamic polarization curve shown in Fig. S5) are possibly ascribed to the chloride-induced non-uniform dissolution of the matrix, or even are believed to result from the breakdown of passive film or meta-stable pitting process/repassivation process. Herein, the measurements of potentiodynamic polarization were only used for determining a suitable passivation potential for the potentiostatic passivation treatment.

It is worthwhile to mention that the samples for the present TEM observation are not those experienced potentiodynamic polarization but those for potentiostatic passivation. During potentiostatically passivating, the current transients usually appear after an induction period. For the passivation time of 30 min adopted in our experiment, breakdown events had not

occurred, which is confirmed by the fact that no any current transient appears in the I-t curve (Fig. S6). In case of current transient appeared, the corresponding specimen was discarded ensuring any possible breakdown events were excluded.

Questions/comments (4): the arguments for eliciting a stress related film breakdown model are weak- and not well described.

Response to Questions/comments (4): We agree with the referee that a stress-elicited film breakdown should be described in more details. It was proposed in some breakdown models that the passive film can be damaged with the assistance of stress within the passive film or at the interface of metal/film. In the SEM images in the literature (such as, J. A. Richardson and G. C. Wood, *J. Electrochem. Soc.* 120, 193 (1973).), ruptured fragments of the passive film were observed in the vicinity of a pit, suggesting that the film at the pit site was more likely damaged by some sort of mechanical force, which motivated a consideration of the origin and role of "stress". By means of STM, Y. Xu *et al* (*J. Electrochem. Soc.* 140, 3448-3457 (1993)) revealed that microscopic roughness inherently exist at the smoothest of metal surface. They used mathematical modeling to analyze the distribution of the compressive electrostatic stress produced by the electric field, indicating that the passive film on the concave region of the surface is subject to high-than-average electrostatic pressure and is, therefore, a preferred site for film rupture, as shown in figure R2 below.

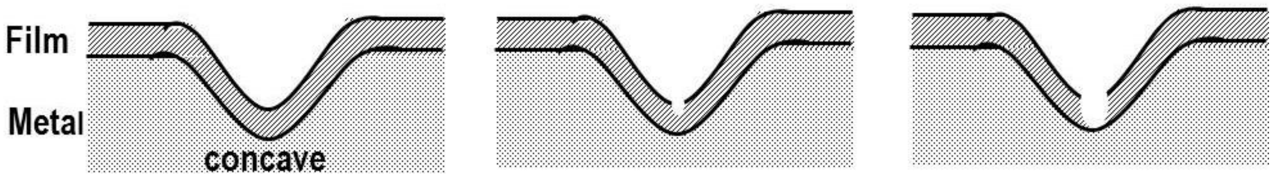

Fig. R2 Schematic maps illustrating the concave site at rough surface is subject to high-than-average electrostatic pressure produced by electric field and is a preferred site for film rupture (Y. Xu, M. H. Wang, and H. W. Pickering, *J. Electrochem. Soc.* 140, 3448-3457 (1993)).

In addition to the undulating surface, in an earlier study based on PDM (Point Defect Model), the undulating interface was proposed, as shown in figure R3 below. It was proposed that the film breakdown would occur at the site of convex interface under the effect of stress (S. Ahn, H. S. Kwon, and D. D. Macdonald, J. Electrochem. Soc., 152, B482 (2005)).

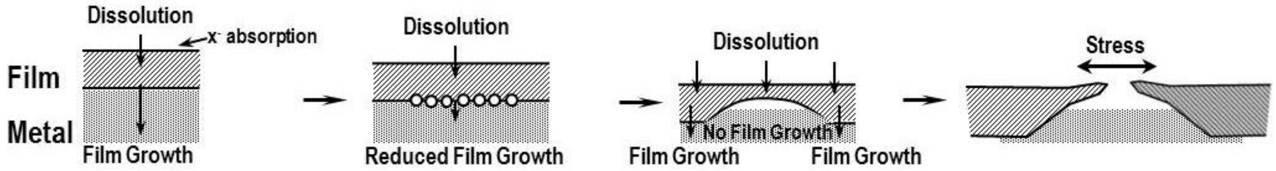

Fig. R3 Schematic maps illustrating stages of undulating interface formation and passive film breakdown occurring at the site of convex interface under the effect of stress according to the PDM (S. Ahn, H. S. Kwon, and D. D. Macdonald, J. Electrochem. Soc., 152, B482 (2005)).

All the studies mentioned above imply that “stress” might exist within the passive film or at the interface site. However, the so-called undulating interface had never been experimentally observed and the stress had never been examined.

In our present work, the as-prepared single crystal matrix provides a distinct metal/film interface and thus enables us to directly observe the interfacial undulations. More importantly, it also provides us an opportunity to examine the strain state within the matrix side using a well-established LADIA simulation method based on the high resolution HAADF-STEM images, since the local lattice distortion immediately below the interface would be induced if a stress exists at the interface.

Although the local strain state that we have extracted is along the direction parallel to the interface of metal/oxide and meanwhile perpendicular to the viewing direction (Figure R4a and 4b), the strain state along the viewing direction is also the case, since these two perpendicular directions are crystallographically equivalent, as shown in Fig. R4c. So, the local strain state is in two-dimensions parallel to the metal/film interface.

In the text, we have concluded that the undulating interface formed in chloride-containing electrolyte shows clear evidence of strain-induced lattice expansion on the matrix side (Fig. 6b). Accordingly, the action of the chloride ions imparts pronounced tension on the passive film along the direction parallel to the interface. When the convex site is getting close to the outer surface of the passive film, the thickness of the passive film becomes quite thin. We rationalize that, under the assistance of the stress, the film therein would be pulled apart and finally a breakdown would occur.

Detailed description has been added in this revised text, as seen in the page 7-8, “Interface Strain Distribution Modeling” section, and 4th paragraph of page 10.

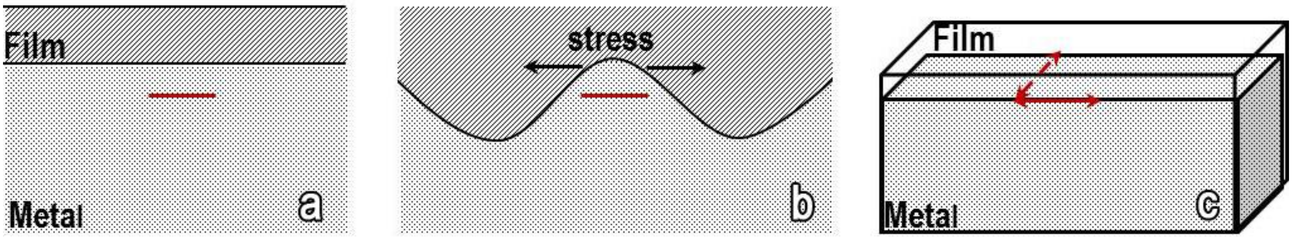

Fig. R4 Schematic maps illustrating the direction (red line) along which local expansion/contraction of next-neighbor atom column distances is modulated. (a) Straight interface formed in chloride-free electrolyte reveals no signs of lattice distortion on the matrix side; (b) Undulating interface formed in chloride-containing electrolyte shows clear evidence of strain-induced lattice expansion. Correspondingly, the tension at the interface is marked with a pair of arrows. (c) Three-dimensional schematic map illustrating the strain state along the viewing direction (a pair of dashed arrows) is equivalent to that as shown in (b).

In addition to the above four major concerns, the referee also raises two minor considerations. We now address these considerations one-by-one in the following.

Minor considerations (1): There is no discussion of possible film damage during sample preparation – or beam induced effects?

Response to Minor considerations (1): We appreciate the referee's consideration of possible film damage of the sample. Indeed, due to the extremely thin passive film with a thickness of only a few nanometers, ensuring the film free of damage during sample preparation and TEM observation is of critically important. As a research team with decades-long experience in transmission electron microscopy, we have numerous skills in guaranteeing what we have observed reflects correctly the original state of the sample.

In the present study, after the cuboid (1.3*2.2*1.5 mm) specimens were polished electrochemically, the surfaces were strictly free of touch in the subsequent sealing, passivating, rinsing, sealing-tape removing steps. During bonding the two passivated surfaces of two samples face-to-face, slip between the two surfaces was avoided in fixation. After the cross-sectional specimen was thinned by grinding, we gave up the dimpling step and directly performed the ion-milling. By doing so, mechanical damage would be most possibly avoided.

The passive film is indeed less resistant to beam irradiation. During the first stage of TEM operation, optical alignment and aberration adjustment were performed at the location far away the target area. Generally, the most serious damage would be at the EDS-mapping experiment during which a large number of scanning points are necessary for ensuring the high resolution in composition distribution, which would yield long time beam irradiation to the film. However, in our experiment, we use the advanced Super-X EDS system with four detectors for the mapping analysis, which extremely shortens the experimental span and thus effectively avoids the beam damage to the film.

We appreciate the referee's kind reminder and we have added these detailed information into the "Supplementary Material" file, please see S1.1.4.

Minor considerations (2): The passivation potentials chosen were very high -why did the authors choose this value?

Response to Minor considerations (2): According to the potentiodynamic polarization curves shown below (Figure R5, also shown in Fig. S5), the $FeCr_{15}Ni_{15}$ single crystal behaves a wide passive region ranging from 100 mV/SHE to 1140 mV/SHE in the chloride-free 0.5 mol/L H_2SO_4 electrolyte. Usually, the representative potentials (in the passive region) include the lower one closed to the active-passive transition region, the middle one located in the middle of the passive region, and the higher one closed to the transpassive region. In the present work, we chose the middle potential of 640 mV/SHE to be the passive film formation potential. Actually, the choosing of such a value is also based on a long-run

perspective consideration. In our next research proposal, we plan to address the effect of passivation potential on the film structures, in which the other distinctive potentials (i.e. lower and higher, as marked with the red arrows in the figure R5) will be a variable parameter.

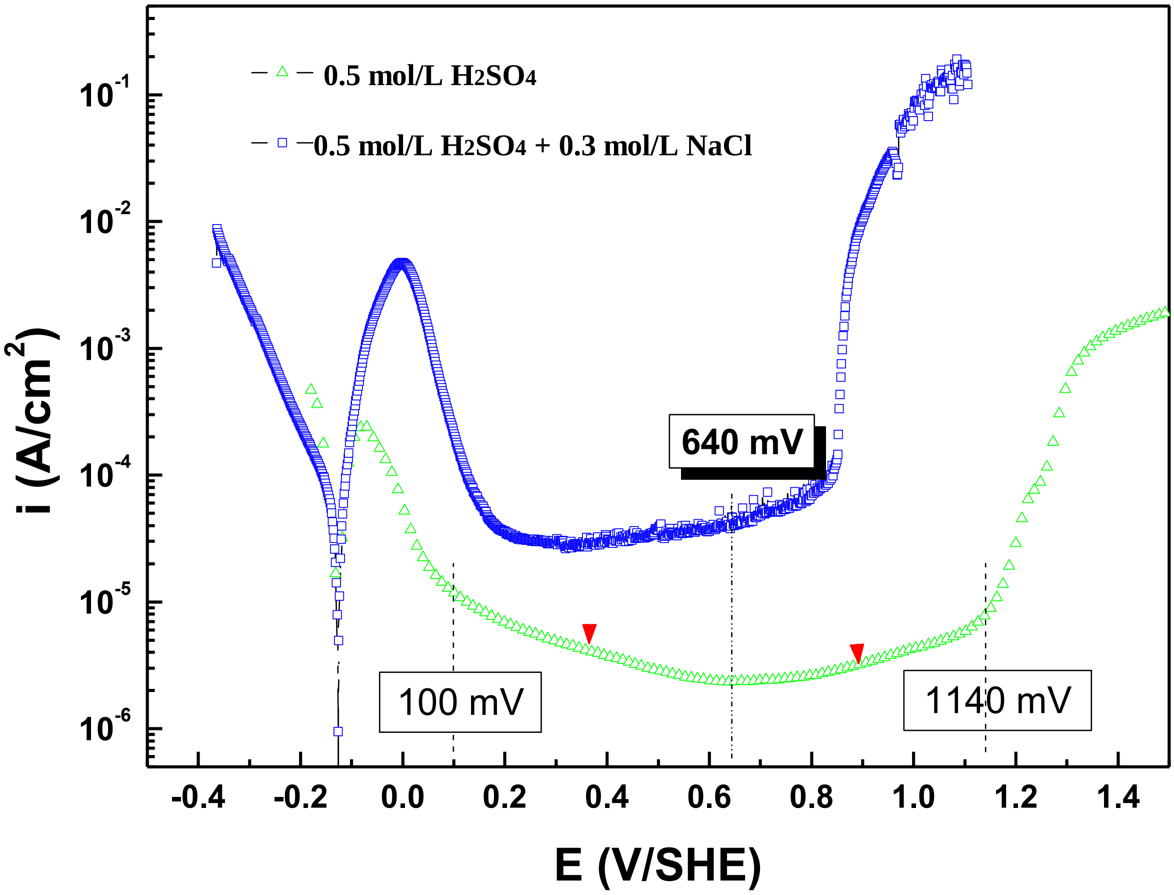

Fig. R5 Potentiodynamic polarization curve of FeCrNi in 0.5 mol/L H_2SO_4 and 0.5 M H_2SO_4 + 0.3mol/L NaCl electrolytes.

Reply to referee #2:

We appreciate the positive comments raised by the referee that" This is a well-researched project that offers clear evidence for the role chloride plays in the initiation of localized corrosion processes on Fe-Ni-Cr alloys", and that "I recommend the paper be published once the following issues have been addressed".

Here, we address all the issues raised by the referee one-by-one in the following.

Issue #1: Since it was conducted on single crystals, the study, while directly addressing how chloride may permeate a passive oxide layer, obviously avoids dealing with the many more likely micro/macro scale defects at which localized corrosion is most likely to occur. This should be made clear in the manuscript.

Response to the issue #1: We appreciate the kind suggestions by the referee that the advantage of conducting the experiments on a single crystal should be emphasized. Indeed, in order to avoid the "the weakest sites breaking down the soonest" which makes figuring out the intrinsic mechanism complex, we grew a $FeCr_{15}Ni_{15}$ single crystal with single austenitic phase. The purpose of growing the single crystal was to make our sample free of any inclusions and grain boundaries. By doing so, the passive films are of high quality with a continuous coverage on the alloy matrix. In addition, with the help of X-ray diffraction, we exposed the low-indexed (001) and (110) plane, respectively, of the single crystal, and performed the anodic polarization. Based on the above designation of single crystal and observations at the passive stage, the TEM imaging in our present study is believed to be able to reflect the nature of how chloride ions interact with the passive film. And in such a case, no site would have already broken down when we try to detect the chloride ions in this study. Detailed information on the $FeCr_{15}Ni_{15}$ single crystal and passive film formation is provided in Supplementary S1.1.2 and S1.1.3.

We appreciate the kind reminder of the referee. A brief description has been added in this revised text (page 3, line 23-27).

Issue #2: While it is the chloride which reaches the alloy/oxide interface that damages the interface leading to the features eventually causing breakdown locations, one would expect to see a gradient of chloride ions throughout the oxide. Is there any evidence that this is the case?

Response to the issue #2: In the EDS maps (Fig. 2 in the main text), we see that chloride concentrate to the inner layer showing the relatively brighter contrast in the image; in the meanwhile, chloride is also incorporated in the outer layer but with lower concentration exhibiting the darker contrast compared with that of the inner. We try to qualitatively display the variety of the chloride density across the film by digitizing the EDS maps (as seen in Figure R6a, which is a part of Figure 2 in the main text). Figure R6b displays the distribution of chloride density across the film. Each data point (Cl mass%) in Figure R6b is extracted

through the area enclosed by a rectangle of 0.12 nm * 30nm, namely the average content, as illustrated in Figure R6c. Every two data points are 0.35nm apart from each other. Indeed, a gradient of chloride ions across the film is remarkable, as the referee proposed.

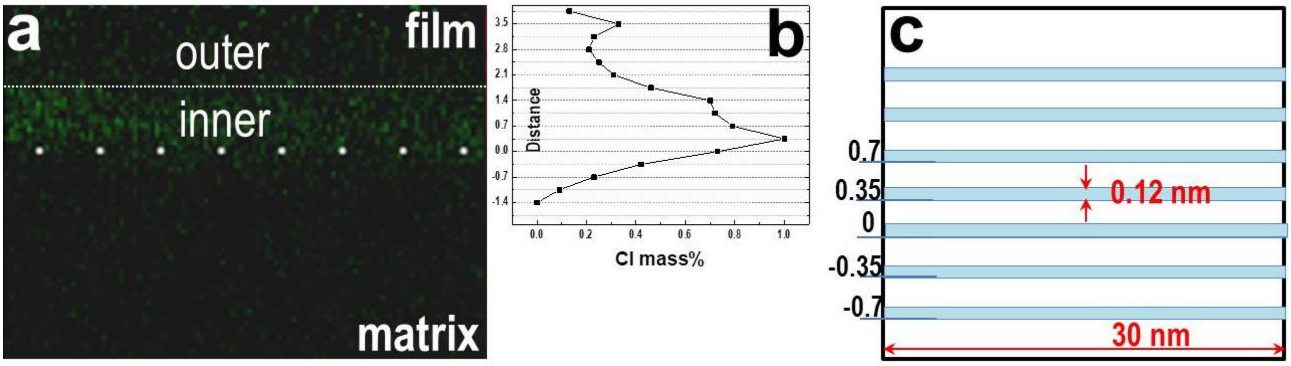

Fig. R6 The variety of the chloride density across the film by digitizing the EDS maps.

Issue #3: The authors state that only specimens which did not exhibit transients were examined. This suggests they are ignoring a primary pathway by which breakdown occurs. The frequency and importance of such transients should be discussed.

Response to the issue #3: We appreciate the concerns raised by the referee. As we know, even the specimen is potentiostatically polarized in a chloride-containing electrolyte within the passive region, it is still in risk of breakdown of passive film and a subsequent repassivation process, which is usually reflected by the current transients in the i-t curve. The frequency of the transients represents the number of the breakdown-repassivation events.

In our study, we avoid an observation after the film had been broken down, this is because once a breakdown occurs, the metal would immediately exposure to the solution and the chloride ions might directly attack the metal. In such a case, it is impossible to identify the history of chloride-attacking upon the passive film. In other words, an observation after breakdown may make us miss the primary information on what really happened in terms of how and where chloride attacks the film leading to the breakdown. This is the reason why in our study we monitor, on the specimens free of any attack (no current transients), the presence of the chloride and the chloride-induced microstructural evolution. We particularly focus on the microstructure evolution induced by Cl^-, and try to directly figure out the weakest site resulting from the chloride attack. We find the chloride-induced undulating interface and the resultant inhomogeneity of the films thickness. And we show how the film is getting thinner and how the weakest site is getting to appear, which is believed to be the preferential site of a breakdown.

We believe that the above information is able to veritably reflect the primary pathway by which breakdown occurs, as schematically illustrated in Figure 7 in the main text file.

In this revised manuscript, we have added this discussion into the "Supplementary Material" file, please see S1.1.3.

Issue #4: The evidence for, and rationale of why, the alloy/oxide adopts an uneven structure are persuasive. However, the arguments offered to explain why these undulations (concave/convex locations) are amplified until breakdown occurred, as schematically illustrated in Figure 7(b), are not totally convincing. Since the interface is more aggressively attacked at locations which initially become concave, leading to a thicker oxide coverage, one might expect these locations to eventually corrode more slowly. As the oxide thickened and the locations of SROs in this new oxide occur, would not the interfacial attack shift to the more convex locations leading to a general surface roughening rather than the enhancement of the undulations? On pages 8 to 10 the authors seem to argue both for and against such a roughening. This discussion needs to be clarified.

Response to the issue #4: We propose that inhomogeneous adsorption of chloride ions on the bare metal surface results in the initial concave and convex interface. As the oxide film gets to thicken progressively, whether or not the concave sites can keep to be more aggressively attacked yielding a faster film growth and the convex sites more gently attacked leading to a slower film growth, is decided by the facility of chloride ion permeation. That depends on two aspects: one is *electric field* and the other is *intrinsic nature of the new oxide film*. In the text of pages 8 to 10, we mainly discuss the effect of electric field that depressing the amplitude of the interface undulation, namely, against such a roughening. The concave interfaces in the film are notably thicker and possess a weaker electric field than the convex interface, and hence pose a greater resistance to chloride ion permeation. Correspondingly, the parts of the metal substrate directly beneath the concave positions in the undulating film would experience/suffer less severe cation injection process, which retards film thickening.

Nevertheless, if the intrinsic nature of the thicker passive film located at some concave interfaces facilitates the chloride permeation, namely, it is less compact or exhibits more connected paths created along the SRO/amorphous, those concave sites can be expected to still keep a faster film growth, and *vice versa* for the convex interface. The cumulative impact of the occurrences ought to amplify some undulations (concave/convex locations), and the convex interfaces have the tendency to move more and more closer to the outer surface of the passive film, as illustrated in Fig. 7b.

According to the large amount of observations in this study, we do find that the rule of interface roughening is quite non-uniform. In some locations, the roughening of interface is very considerable, as shown in the HAADF-STEM image below (Figure R7a, also shown in Fig. S12). In the zoom-in image (b) which is enlarged image of the area marked with a rectangular in (a), the convex site at the matrix side (featuring with the well-defined lattice images) is getting close to the outer surface of the passive film, wherein, the thickness of

the passive film is quite thin. It is reasonable to propose that, with further roughening, the film therein would be thinner and thinner and finally a breakdown would occur.

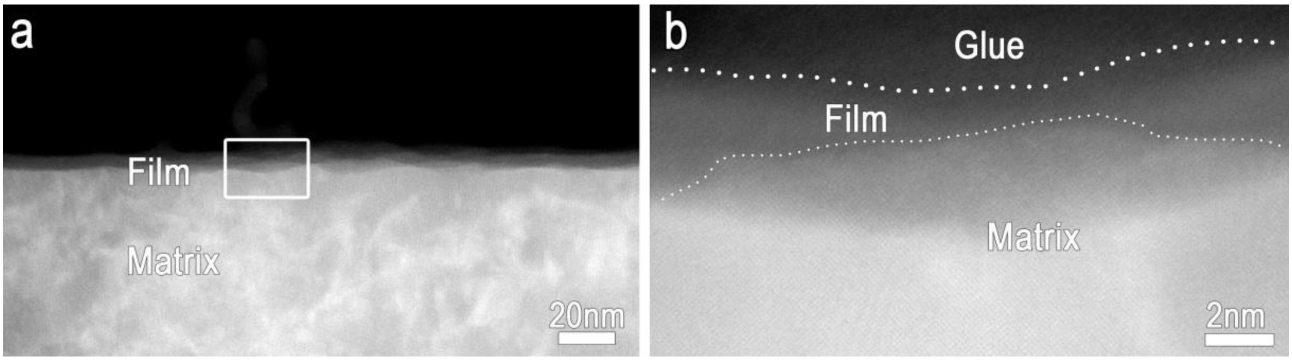

Figure R7 Chloride-induced large roughening at the metal/passive film interface. Image in (b) is an enlargement of the area marked with a rectangular in (a).

Pit nucleation, initiated at the surface of high purity metals or even of single crystals, is generally known as random and unpredictable. Our present experimental results and the analysis above indicate that convex sites where the nature of the passive film facilitates its amplification would be the preferential sites for film breakdown and pit nucleation.

In this revised text, we have made some modification to clarify the correlated discussion (line 25-33 of page 9, line1-12 and line 27-32 of page 10, and line 1-2 of page11).

Issue #5: The transfer of metal ions across the alloy/oxide interface is described as dissolution even when the oxide is well-developed. This does not seem an appropriate description at this interface. The term "cation injection" might be better.

Response to the issue #5: We fully agree with the referee, and here we have replaced the word "dissolution" by "cation injection" in the revised manuscript, as seen in Page 9-11.

In addition to the above five major issues, the referee also raises seven minor ones. We now address these minor issues one-by-one in the following.

Minor issue #1: Many of the paragraphs are up to, and even over, 1 page long, which makes the manuscript tedious to read in places.

Response to the minor issue #1: We appreciate the kind criticism raised by the referee. Indeed, the paragraphs on "Passive Film Structure and Chloride Ion Interactions (page 3-7)" and "Chloride Ion Attack Mechanism (page 8-11)" are quite long. We now have separated them and in the meanwhile we used subheadings for a better thematic structure.

Minor issue #2: Presumably the oxides were grown in deaerated solutions?

Response to the minor issue #2: All the solutions in which the passive film are anodically grown are not deaerated. This statement is added in the S1.1.3 section in the Supplementary Materials.

Minor issue #3: In the legend to Figure 3, it should be stated that the yellow line indicates the alloy/oxide interface. In Figure 3(c) it is not clear that this line faithfully tracks the interface. What criterion was used to define where this line should be?

Response to the minor issue #3: According to the kind reminder of the reviewer, we have added a statement of "the yellow dotted line indicates the metal/passive film interface" in the legend to Fig. 3. The interface can be identified by the boundary between the well-defined lattice image of the metal and the noncrystalline film, as seen in the enlarged micrograph (Fig.R8) of figure 3c. Such a two-dimensional lattice image is obtained by tilting the crystal to [001] crystallographic direction, which could be done easily based on the as-grown single crystal.

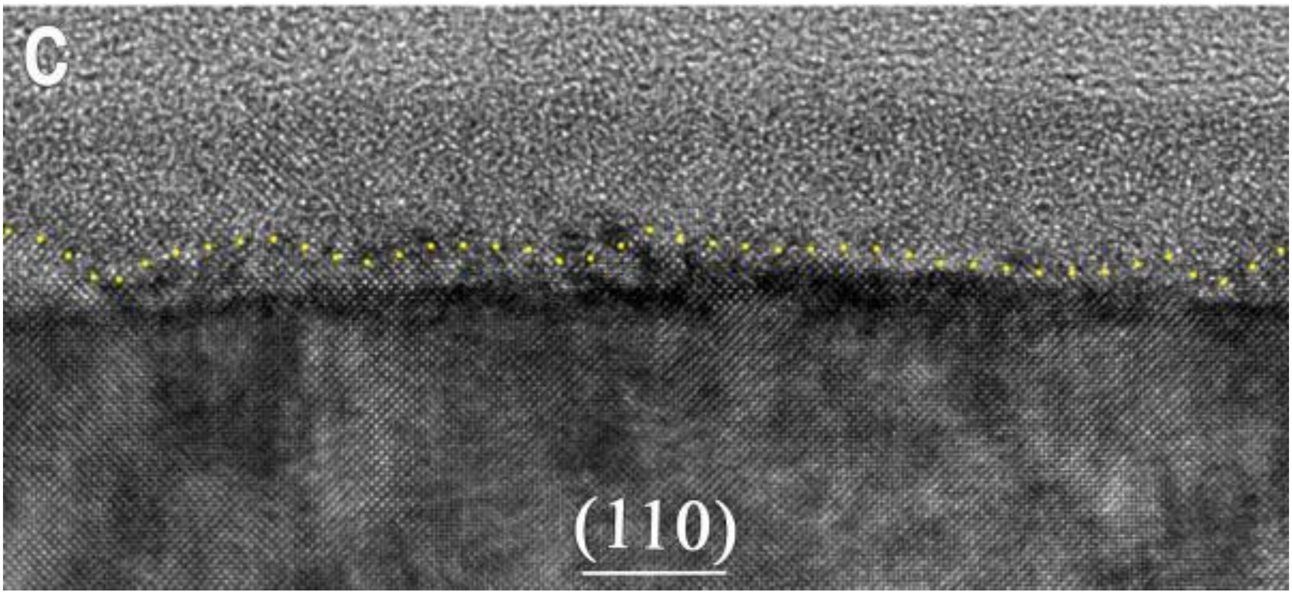

Fig. R8 An enlarged image of Fig. 3c in the text. The yellow dotted line indicates the metal/passive film interface. Immediately beneath the yellow dotted line, the well-defined 2D lattice corresponding to that of the substrate are clearly imaged, whereas the upper area evidently displays the amorphous characteristics of the passive film embedded with SRO structure.

Minor issue #4: On page 6, it is stated that the interfaces between the SRO structures and the amorphous zone assume the features of grain boundaries. The authors should be more specific about the features they allude to.

Response to the minor issue #4: A grain boundary usually features an interface of two grains with different orientations of the same phase; the concept of a grain boundary may also in principle extend to an interface of two phases with different crystal structures. A grain boundary exhibits an irregular atom array, which is different from that of alternative of the adjacent crystalline grains. The irregular atom array yields a loose structure at a grain boundary, thus provides tunnels for species diffusion and transport. In our present study, a SRO structure can be well identified by its 2D periodic lattice image; whereas the amorphous phase always features a random atom distribution. Thus, the "grain boundary" between

SRO and amorphous phase can be readily identified according to the high-resolution TEM images. Nevertheless, a determination of atomic configurations at this kind of boundary must be a big challenge, which should be more complex than that of two crystalline grains. We propose that the SRO/amorphous phase boundary must feature atomic randomness on the one hand, and on the other hand, an atomic-scale elemental redistribution at the outmost layer of SRO structure might be also possible. Whatever the specific atomic structure features, grain boundaries, whether referred to boundaries between crystalline oxide grains or to boundaries of SRO/amorphous phase, usually provide tunnels for species diffusion and transport yielding less resistant to Cl- ion attack.

Based on the above discussion, we have added some statement on the features of boundary between SRO structures and the amorphous zone, as seen in page 6, line 10-13 and line 24-30.

Minor issue #5: The significance of the colour bar in Figures 6 and S10 needs to be specified.

Response to the minor issue #5: The colour bar on the right in Fig. 6 and S10 indicates the normal strain, where the positive values in the colour bar represent tensile strain and negative values indicate compressive strain. The signification of the colour bar has been added in the legend to Fig. 6 and S10.

Minor issue #6 (for supplementary materials): It is stated that the passive film is ~ 3nm thick. This seems at odds with the TEM image in Figure S4 in which the film looks significantly thicker.

Response to the minor issue #6: We appreciate the reminder by the referee. This is a typing error in the first page of supplementary materials. As seen in page 4 of the main text, "Aberration-corrected transmission electron microscopy (Cs-corrected TEM) revealed a passive film with thickness of about 4-5 nm (see TEM image in Fig. S4)". The thickness of the film shown in Figure S4 is estimated to be approximately 4.2 nm. We have revised this error in the revised version of supplementary materials.

Minor issue #7 (for supplementary materials): "The specimen was depolarized at -1.2V for 30s before potentiostatic passivation which avoided the native oxide formation in air". While this is a standard treatment, there is evidence that such native oxides are not removed, only rendered highly defective. The authors should comment on this and whether it would affect their study.

Response to the minor issue #7:

We appreciate the concern raised by the referee, and we are confidential that such a standard treatment does not affect our conclusion.

Usually, before an electrochemical measurement, such as potentiodynamic or potentiostatic polarization, depolarization at a cathodic potential is recognized to be a standard treatment.

By the cathodic reduction, the native oxide is generally believed to be stripped. Nevertheless, extensive investigations focusing on the nature of the chloride interactions with the oxide film using XPS and AES techniques actually indicate that the cathodic reduction pre-treatment could affect the observed results on an incorporation of chloride in the passive oxide film. Natishan et al[1] examined the specimens potentiostatically passivated in the chloride-containing solution and failed to detect the Cl^- adsorption or incorporation into the passive film, which appears to be contradictory with the result in some other studies on Cl^- in the oxide [2-7]. The only difference in these studies is that the native oxide was stripped cathodically prior to passivation in the previous experiments whereas it was not in Natishan's study. Natishan et al also added the pre-treatment of cathodically stripping the native oxide in chloride containing solution and then re-examined the samples. They found Cl^- incorporation in the passive film, and thus concluded that retaining the native oxide followed by passivation in a Cl-containing solution was sufficient to prevent subsequent Cl^- adsorption or incorporation. The defense effect of the pre-formed oxide film was further approved by the evidence that Cl^- was not detected in the samples undergoing a pre-treatment of cathodic depolarization followed by passivation in a Cl-free solution (forming a passive film anodically)[8-14]. In summary, previous studies actually imply that the extent of Cl^- incorporation in the passive film could depend on a specific presence of pre-existed oxide film on the metal (corresponding to the cathodic depolarization),-native oxide, or anodic oxide.

Certainly, it is also evidenced that the native oxides can't be removed by cathodic reduction pre-treatment, only rendered highly defective.

In spite of the above various experimental observations, the pretreatment that cathodic depolarization at -1.2V for 30s in our present work would not affect our conclusion on the chloride attack mechanism. This is rationalized by the fact that, in order to monitor the transport and effect of chloride ions, we designated three conditions for the formation of passive films: (1) passivation in H_2SO_4 electrolyte, (2) passivation in H_2SO_4 + NaCl electrolyte, and (3) initial passivation in H_2SO_4 electrolyte and subsequent addition of NaCl into the H_2SO_4 electrolyte. The reason that we designated condition 1 and 2 was to compare and figure out the representative characteristics of the interface attacked by chloride, which should have nothing to do with where the chloride comes from (during film formation or by permeation) and how the chloride incorporation is affected by pre-treatment. Once the chloride-attacked interface is figured out, we are able to conclude the permeation mechanism in condition 3, since we find rather similar interfacial phenomena between condition 2 and 3. These phenomena include the undulating interface and some concentration of chloride, which inhomogeneously occur along the interface. It is noteworthy that, in condition 3, between cathodic depolarization and contact with Cl^-, a passive film was already formed in Cl-free solution. In such an experimental procedure, the incorporation of chloride in the film is irrelevant to the cathodic reduction treatment.

References in Response to the minor issue #7：

1. P.M. Natishan, W.E. O'Grady, F.J. Martin, *J. Electrochem. Soc.* **158,** C7 (2011).

2. B. MacDougall, D. Mitchell, G. Sproule, M. Graham, *J. Electrochem. Soc.* **130**, 543 (1983).

3. I. Olefjord, B. Brox, and U. Jelvestam, *J. Electrochem. Soc.* **132**, 2854 (1985).

4. A. R. Brooks, C. R. Clayton, K. Doss, and Y. C. Lu, *J. Electrochem. Soc.* **133**, 2459 (1986).

5. I. Olefjord and L. Wegrelius, *Corros. Sci.* **31**, 89 (1990).

6. S. Mischler, A. Vogel, H. J. Mathieu, and D. Landolt, *Corros. Sci.* **32**, 925 (1991)

7. S. Ningshen, U. K. Mudali, V. K. Mittal, and H. S. Khatak, *Corros. Sci.* **49**, 481 (2007).

8. W. Khalil, S. Haupt, and H. H. Strehblow, *Werkst. Korros.* **36**, 16 (1985).

9. R. Goetz, B. MacDougall, and M. J. Graham, *Electrochim. Acta* **31**, 1299 (1986).

10. V. Mitrovic-Scepanovic, B. MacDougall, and M. J. Graham, *Corros. Sci.* **27**, 239 (1987).

11. D. Landolt, S. Mischler, A. Vogel, and H. J. Mathieu, *Corros. Sci.* **31**, 431 (1990).

12. C. Hubschmid and D. Landolt, *J. Electrochem. Soc.* **140**, 1898 (1993).

13. C. Hubschmid, D. Landolt, H.J. Mathieu, *Fresenius J. Anal. Chem.* **353**, 234 (1995).

14. C. O. A. Olsson and D. Landolt, *Electrochim. Acta* **48**, 1093 (2003).

Reply to referee #3:

We appreciate the general recognition by the referee that "The research topic is of great importance itself, while certainly of involving complicated factors that often are hard to be sorted out", and that "the TEM observation of passivation film structure and chemistry appears to be solid". Nevertheless, the referee also raises some comments which can be summarized into two aspects.

The first comment raised by the referee is that: "The focus of this work appears to be on the corrosion mechanism of FeCr15Ni15 alloy. Checking into the details of the present work, it is apparent that the authors have captured some details regarding the structure and chemistry of the passivation film. Taken from there, the authors have attempted to interpret the accelerated corrosion with the presence of chlorine, and from that point, the manuscript appears to be driven toward divergence, with apparent mishandling of fundamentals on corrosion science".

The second comment raised by the referee is that: "The general conclusion regarding the chlorine transport in the amorphous region between the crystallized domains would be different from that of a whole amorphous phase is apparently a wrong one".

Regarding the first comment, we would like to clarify that the present work does not focus on the corrosion mechanism of $FeCr_{15}Ni_{15}$ alloy, instead, we focus on the atomic-scale mechanism of chloride attack on the passive film of metals. For this objective, firstly we design and grow a single crystal of $FeCr_{15}Ni_{15}$ which enable us to obtain a distinct metal/passive film interface and better characterize the structure of the interface region as well as the chloride-induced structural evolution; and second, we perform the advanced aberration-corrected transmission electron microscopic observations, during which high-resolution imaging and Super-X EDS analysis are extensively used.

Our study definitely does not mishandle the fundamentals on corrosion science, but delivers new information which is never known before. We have directly observed that the chloride ions actually permeate not only the outer but also the inner layer of the films to attack the interface. We find the chloride-induced structural evolution at the metal/film interface, including the chloride-induced lattice expansion on the metal side, interfacial undulations and structural alterations to the film. And we show how the film is getting thinner and how the weakest site is getting to appear, leading to the preferential site of a breakdown.

The significant contribution of the present study is remarkable, as confirmed in the comments raised by the other three referees that "the experiments are challenging and the data appear robust", that "This is a well-researched project that offers clear evidence for the role chloride plays in the initiation of localized corrosion processes on Fe-Ni-Cr alloys", that "the experimental work is of high quality and makes a convincing story", and that "the key experimental claims of the paper are all very significant and worthy of publication".

Indeed, there are several classic theories on the interaction of chloride and passive film, but agreement has not been made in these models. The key reason is believed to result from the fact that many of them are based on hypotheses and theoretical models, and oftentimes, neither sufficiently nor directly corroborated by experimental evidences. To make the arguments come to the end, direct observations, showing the location of chloride appearance and the chloride-induced structural evolution in the film particularly at the interface, should be the key. We expect that our findings in the present study would stimulate the scientists in this community to reacquaint the atomic-scale mechanism of passivity breakdown on various metals.

Regarding the second comment, we would like to clarify that we never concluded that "the chlorine transport in the amorphous region between the crystallized domains would be different from that of a whole amorphous phase". Instead, we emphasize that the interface between a SRO structure and an amorphous zone be a special kind of grain boundaries and thus provide a ready path for the chloride ion transport. Namely, when chloride ions attack the as-grown passive film, chloride ions only get to certain interfacial locations by heterogeneously penetrating the as-grown film along the connected path provided by the interfaces between SRO structures and the amorphous zone. All of our experimental results indicate clearly that chloride ions remarkably and unilaterally modify the interface zones via lattice expansion on the metal side, and induced undulations at the interface and structural inhomogeneity on the film side. Such a series of events, by which chloride ions incorporate and attack the passive film, were neither envisaged nor considered in the available theories describing chloride-induced passivity breakdown.

Reply to referee #4:

We appreciate the positive comments and recommendation by the referee that "the experimental work is of high quality and makes a convincing story", and that "The key experimental claims of the paper, concerning the accumulation of chloride ions at the metal/film interface, the distortion of the interface and the strain in and local structure of the amorphous oxide are all very significant and worthy of publication". However, the major thing complained by the referee is that "the theoretical analysis does not match the quality of the experimental work".

We fully understand the referee's expectations, which can be summarized basically into four concerns. Here we try to address all these concerns one-by-one in the following.

Concern #1: There are major conclusion drawn from the literature (first paragraph) that are boldly asserted and not supported by any analysis.

Reply to the concerns #1: The statement in the first paragraph that the reviewer referred should be "Of the existing hypotheses, there are two divergent schools of thought: One advocates that the chloride ions, under the influence of the electric field and/or by means of oxygen vacancies, permeate the passive film to reach the metal/oxide interface. The other postulates that the chloride ions do not penetrate the passive film, but are rather absorbed at the barrier/outer layer interface in the film."

Actually, we authors want to deliver such an idea that, whether or not Cl^- incorporating and the accurate location in the passive film are crucial issues for clarifying the exact nature of the chloride interactions with the passive film leading to film breakdown. In the enormous existing experimental data addressing these issues, much of evidence on the incorporation of Cl^- in the passive oxide film on Fe, Ni, Fe-Cr alloys or stainless steels can be mainly classified into two groups: one is chloride incorporation[1-10], and the other is chloride absence in the passive film [6,11-17]. In the case of incorporation, the location of chloride in the passive film is also controversy. Some investigators identified Cl^- to be located or concentrated in the outer layer of the film[1-6], whereas some others in the inner layer[7,8]. Accordingly, the models describing the film breakdown include, not limit to, two types of interaction of chloride with the film: penetration and adsorption.

The reviewer's comments remind us of the fact that the statement in the previous version might not be the best way to emphasize our real attention. So, we have rewritten this paragraph based on the above analysis, as seen in the 2nd paragraph of page 2.

References in Reply to the concerns #1:

1. D. F. Mitchell, Appl. Surf. Sci. **9**, 131 (1981).

2. B. MacDougall, D. Mitchell, G. Sproule, M. Graham, *J. Electrochem. Soc.* **130**, 543 (1983).

3. I. Olefjord, B. Brox, and U. Jelvestam, *J. Electrochem. Soc.* **132**, 2854 (1985).

4. A. R. Brooks, C. R. Clayton, K. Doss, and Y. C. Lu, *J. Electrochem. Soc.* **133**, 2459 (1986).

5. S. Mischler, A. Vogel, H. J. Mathieu, and D. Landolt, *Corros. Sci.* **32**, 925 (1991)
6. C. Hubschmid, D. Landolt, H.J. Mathieu, *Fresenius J. Anal. Chem.* **353**, 234 (1995).
7. O. Murphy, J.M. Bockris, T. Pou, L. Tongson, M. Monkowski, *J. Electrochem. Soc.* 130, 1792 (1983).
8. I. Olefjord and L. Wegrelius, *Corros. Sci.* **31**, 89 (1990).
9. P. Marcus, J.M. Herbelin, *Corros. Sci.* **34**, 1123 (1993).
10. S. Ningshen, U. K. Mudali, V. K. Mittal, and H. S. Khatak, *Corros. Sci.* **49**, 481 (2007).
11. W. Khalil, S. Haupt, and H. H. Strehblow, *Werkst. Korros.* **36**, 16 (1985).
12. R. Goetz, B. MacDougall, and M. J. Graham, *Electrochim. Acta* **31**, 1299 (1986).
13. V. Mitrovic-Scepanovic, B. MacDougall, and M. J. Graham, *Corros. Sci.* **27**, 239(1987).
14. D. Landolt, S. Mischler, A. Vogel, and H. J. Mathieu, *Corros. Sci.* **31**, 431 (1990).
15. C. Hubschmid and D. Landolt, *J. Electrochem. Soc.* **140**, 1898 (1993).
16. C. O. A. Olsson and D. Landolt, *Electrochim. Acta* **48**, 1093 (2003).
17. P.M. Natishan, W.E. O'Grady, F.J. Martin, *J. Electrochem. Soc.* **158,** C7 (2011).

Concern #2: The section on chloride ion attack mechanism is really just a restatement of the observed experimental points without any substantive theoretical enhancement or analysis. Similarly in the interface strain distribution modelling the authors make bold claims without analytical justification - e.g chloride ions impart tension that can lead to film breakdown. The later statement is very important and may be true but is not justified.

Reply to the concerns #2: We really appreciate the expectations raised by the referee, and, we try our best to add more analysis to the experimental identifications. The most significant experimental results are the chloride-induced undulating interface and the expansion of the lattice in matrix. In the section of 'chloride ion attack mechanism', we analyze the means by which chloride remarkably modify the interface zones. We also discuss how the undulating interface is formed and amplified, and how the lattice expansion is induced by chloride. Meanwhile, the convex sites are proposed to be the preferential locations of passive film breakdown with assistance of the interfacial tension. Accordingly, we further propose that the film breakdown sites are not really locations with high chloride ion concentration (concave sites) as widely believed, but are actually the adjacent locations where the effect of chloride ions is relatively weak (convex sites). All these points are indeed new understanding on the chloride attack on the passive film at atomic scale. Actually it is not a simple restatement of the observed experimental points.

In this revised manuscript, the original 'chloride ion attack mechanism' section has been modified to be "New Understanding on Chloride Attack on the Passive Film at Atomic Scale", wherein, two issues was emphasically discussed with subtitles of '*Formation and amplification of undulating interface under effect of chloride*' and '*Chloride-induced lattice expansion to the matrix*' (page 8-11). We believe that the discussion on the chloride attack on the passive film is more clarified after the rewriting.

To justify film breakdown by chloride ions impart tension, in this revised manuscript we

made more discussions and also summarized below.

In our present work, the as-prepared single crystal matrix provides a distinct metal/film interface and thus enables us to directly observe the interfacial undulations. More importantly, it also provides us an opportunity to examine the strain state within the matrix side using a well-established LADIA simulation method based on the high resolution HAADF-STEM images, since the local lattice distortion immediately below the interface would be induced if a stress exists at the interface.

Although the local strain state that we have extracted is along the direction parallel to the interface of metal/oxide and meanwhile perpendicular to the viewing direction (Figure R9a and R9b), the strain state along the viewing direction is also the case, since these two perpendicular directions are crystallographically equivalent, as shown in Fig. R9c. So, the local strain state is in two-dimensions parallel to the metal/film interface.

In the text, we have concluded that the undulating interface formed in chloride-containing electrolyte shows clear evidence of strain-induced lattice expansion on the matrix side (Fig. 6b). The lattice expansion in the matrix side means a tensile strain at the interface. Correspondingly, a pronounced tension along the direction parallel to the interface is imparted to the passive film as a result of interface undulating induced by chloride ions. When the convex site is getting close to the outer surface of the passive film, the thickness of the passive film becomes quite thin. We rationalize that, under the assistance of the stress, the film therein would be pulled apart and finally a breakdown would occur. Such a direction of stress has been labeled in the revised Fig. 7 (also seen in Figure R10).

Detailed description has been added in this revised text, as seen in the page 7-8, "Interface Strain Distribution Modeling" section, and 4th paragraph of page 10.

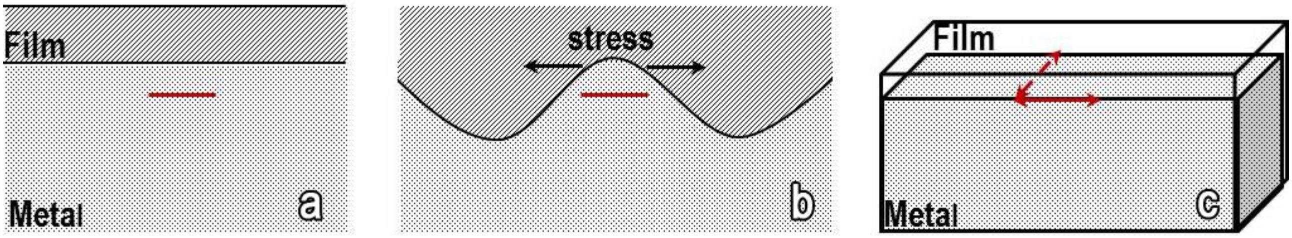

Fig. R9 Schematic maps illustrating the direction (red line) along which local expansion/contraction of next-neighbor atom column distances is modulated. (a) Straight interface formed in chloride-free electrolyte reveals no signs of lattice distortion on the matrix side; (b) Undulating interface formed in chloride-containing electrolyte shows clear evidence of strain-induced lattice expansion. Correspondingly, the tension at the interface is marked with a pair of arrows. (c) Three-dimensional schematic map illustrating the strain state along the viewing direction (a pair of dashed arrows) is equivalent to that as shown in (b).

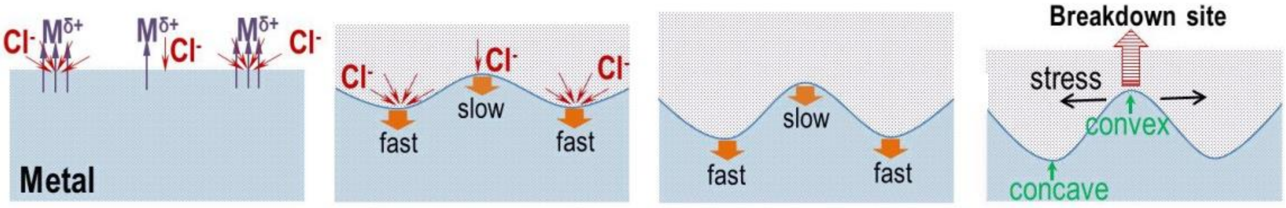

Figure R10 In chloride-containing (0.5 mol/L H_2SO_4 + 0.3 mol/L NaCl) electrolyte, the tendency of chloride ions to be preferentially adsorbed at defective sites yields non-homogeneous adsorption on the bare metal surface. High chloride ion concentration would induce faster dissolution of Fe ions, which leads to inhomogeneous interface-movement rates, hence differences in passive film growth rates. Such a process yields a passive film with an irregular and undulating Me/BL interface.

Concern #3: The problems with the theoretical analysis is confounded by two other factors - the authors do not in any substantive way relate their observations but to the detail of any of the historically postulate mechanism and the reference to both theoretical and experimental developments are in the main part prior to the year 2000.

Reply to the concerns #3: We appreciate the kind analysis raised by the referee. Yes, when we sum up our present study, we always keep in mind of our new finding with reference to previous classic literature. Passivity is a classic issue, and since 1960s numerous theoretical hypothesis and models have been proposed describing the growth and breakdown process of passive film. And some of hypothesis have been continually developed and optimized, leading to well-known or even generally accepted theories though they are only postulates. The lack of agreement on the mechanism of passive film breakdown is mainly due to the difficulty encountered in obtaining precise experimental information. In our present work, we provide *direct* evidence on where the chloride ions are and how they interact with the metal. Our finding is not tandem with some well-known hypothesis in terms of the manner of chloride permeation and the formation of undulating interface. By reviewing the existing theories, we try to discuss the relevance and distinction between our claims and the details of some historically postulate mechanism.

Our experimental findings support the penetration mechanism, but find out the deficiency in the often-cited oxygen vacancy-assisted transport of Cl^- mechanism, which is clearly contrary to the selective permeation identified in our study (page 5-6 in the original submitted text). When discussing the role of SRO/amorphous boundaries in providing tunnels for species diffusion and transport, we also review the related works which accept the roles of grain boundaries in passivity breakdown and initiation of localized corrosion (page 6 in the original submitted text). Also in discussing the possible breakdown sites of the passive film, we refer to the PDM which propose a highly undulating interface (similar to our observation) and the film breakdown at the site of convex interface under the effect of stress, however, the mechanism for the formation of undulating interface in our study is completely different from the previous on the basis of PDM (page 10 in the original

submitted text).

Actually, all the new-understanding on 'where, how and why' chloride attack on the passive film are concluded by relating our direct observations at the atomic scale. And in presenting our observations, we tried our best to involve a fuller set of references to previous relevant work including those in recent 10 years, making the contextualization of the study rich of major work in this field. Regrettably, this kind of presentation was likely confounded by inserting discussions on the details of some historically postulate mechanisms.

According to the comments raised by the referee, in this revised manuscript we have made the "Results" section sub-headed, and re-structured our manuscript by first describing our experimental findings. We also removed the discussions on our finding with the PDM and the often-cited oxygen vacancy-assisted transport of Cl^- mechanism.

Since the chloride-induced film evolution is central issue for addressing the film breakdown, the recent experimental advances promote many novel and powerful evidence on the structure and chemistry of the passive film as well as the evolution imparted by chloride. We have updated the reference list by adding some reference after the year 2000 (Ref. 37, 42-46, 48-52, 54, 57-58 and 60-64 in page 2 of the revised text), and also show below:

References in Reply to the concerns #3:

[1] D. Zuili, V. Maurice, P. Marcus, *J. Electrochem. Soc.* **147,** 1393-1400 (2000).

[2] Y. Nemoto, Y. Miwa, M. Kikuchi, Y. Kaji, T. Tsukada, H. Tsuji, *J. Nucl. Sci. Technol.* **39,** 996-1001 (2002).

[3] E.E. Rees, M.P. Ryan, D.S. McPhail, *Electrochem. Solid-State Lett.* **5,** B21-B23 (2002).

[4] A.T. Fromhold, S.J. Noh, *J. Electrochem. Soc.* **150,** B574-B582 (2003).

[5] M.J. Graham, *Corrosion* **59,** 475-488 (2003).

[6] D.H. Kim, H.H. Lee, S.S. Kim, H.C. Kang, D.Y. Noh, H. Kim, S.K. Sinha, *Appl. Phys. Lett.* **85,** 6427-6429 (2004).

[7] J.C. Rao, X.X. Zhang, B. Qin, K.K. Fung, *Ultramicroscopy* **98,** 231-238 (2004).

[8] M. Murayama, N. Makiishi, Y. Yazawa, T. Yokota, K. Tsuzaki, *Corros. Sci.* **48,** 1307-1318 (2006).

[9] A. Seyeux, V. Maurice, L.H. Klein, P. Marcus, *J. Electrochem. Soc.* **153,** B453-B463 (2006).

[10] R.S. Yassar, L. Scudiero, A.S. Alamr, D.F. Bahr, M.G. Norton, *Thin Solid Films* **518,** 2757-2763 (2010).

[11] D.D. Macdonald, *Electrochim. Acta* **56,** 1761-1772 (2011).

[12] W. Fredriksson, S. Malmgren, T. Gustafsson, M. Gorgoi, K. Edström, *Appl. Surf. Sci.* **258,** 5790-5797 (2012).

[13] J. Högström, W. Fredriksson, K. Edstrom, F. Björefors, L. Nyholm, C.O.A. Olsson, *Appl. Surf. Sci.* **284,** 700-714 (2013).

[14] K. Leistner, C. Toulemonde, B. Diawara, A. Seyeux, P. Marcus, *J. Electrochem. Soc.* **160,** C197-C205 (2013).

[15] M. Salasi, G.B. Stachowiak, G.W. Stachowiak, M.R. Kilburn, *Corros. Sci.* **67,** 298-303 (2013).

[16] S. Fajardo, D.M. Bastidas, M.P. Ryan, M. Criado, D.S. McPhail, R.J.H. Morris, J.M. Bastidas, *Appl. Surf. Sci.* **288,** 423-429 (2014).

[17] D.D. Macdonald, *Corros. Eng. Sci. Techn.* **49,** 143-155 (2014).

[18] H.H. Strehblow, *Electrochim. Acta* **212,** 630-648 (2016).
[19] Y. Li, Y.F. Cheng, *Appl. Surf. Sci.* **396,** 144-153 (2017).

Concerns #4: One minor point in the introduction the authors claim that no technique has looked at the evolution of the passive film etc. This is strictly true but a number of authors have carried out similar studies where they look at passive films at set times in a similar way to the authors.

Reply to the concerns #4: We appreciate the kind reminder by the referee. Yes, several groups have tried to carry out similar studies via TEM, although they failed to provide much new information on passive films because of the low spatial resolution therein. Nevertheless, in this revised manuscript we introduce our work with references including these studies (as seen in page 2 of the introduction section, Ref.37-40 in this revised manuscript).

References in Reply to the concerns #4:

[1] Murayama, M., Makiishi, N., Yazawa, Y., Yokota, T. & Tsuzaki, K. Nano-scale chemical analyses of passivated surface layer on stainless steels. *Corros. Sci.* **48**, 1307-1318, (2006).
[2] E. Hamada, K. Yamada, M. Nagoshi, N. Makiishi, K. Sato, T. Ishii, K. Fukuda, S. Ishikawa, T. Ujiro, *Corros. Sci.* **52,** 3851-3854 (2010).
[3] R. Soulas, M. Cheynet, E. Rauch, T. Neisius, L. Legras, C. Domain, Y. Brechet, *J. Mater. Sci.* **48,** 2861-2871 (2013).
[4] K. Oh, S. Ahn, K. Eom, K. Jung, H. Kwon, Observation of passive films on Fe–20Cr–xNi (x=0, 10, 20 wt.%) alloys using TEM and Cs-corrected STEM–EELS, *Corros. Sci.* **79,** 34-40 (2014).

In summary, we appreciate the rather positive evaluation raised by the referee and we fully understand the referee's expectations on theoretical analysis. According to the referee's suggestions, we have tried our best to rationalize our experimental findings and to unmask the chloride attack mechanism on the metal. Agreeing with the referee, we believe that the present experimental advances are significant for stimulating the scientists in this field to revisit the existing theoretical models by taking the atomic-scale information into account. We also agree with the referee that publishing the work in an experiment-oriented one is a choice as well.

Reviewers' comments:

Reviewer #2 (Remarks to the Author):

The authors have revised the manuscript substantially in response to the reviewers comments. Their response to my comments are appropriate.
While they have not fully convinced me that their proposed mechanism(s) are proven I see no reason to deny publication.

(their remarks to the Authors' response to Reviewer #1)
The authors responses [to Reviewer #1] are adequate.
This paper will not settle all the issues under debate but will add substantially to the on-going discussion.

Reviewer #3 (Remarks to the Author):

The authors take great effort for revising the manuscript, clarifying a lot of confusions. Overall,the work includes some information that may be publishable, while the overall characterization of the film and the explanation appear to be still flawed as detailed in the following.

1) The authors need to take care regarding the terminology. The term of "chloride ion" is not a correct term, I think they refer to "chlorine ion". So, they need carefully check the term of chloride, chloride ions, chlorine, and chlorine ions. Please make sure to use the right term at the right context, otherwise, it will be misleading.

2) The structural nature of the passivation film is not clearly identified. The authors use the term of mostly amorphous, with short range order. This gives an impression of the two phase structure. Scrutinizing at the HRTEM images of Figure 4, one can see the crystal of several nanometers. So the statement of the mostly amorphous structure with short range order is very misleading. I would suggest they use dark field image and electron diffraction pattern to clearly reveal the true structural nature of the film. HRTEM image can be misleading for interpretation of local structure in relevance to the surrounding environment due to thickness variation caused contrast variation.

3) The HRTEM images shows contrast modulations, what the origin of this kind of modulation? is this Moiré fringe (I do not believe that), or something else? Looking into the recent publications on Cu-Au alloy oxidation, it appears similar contrast modulation has been identified to be attributed to the interface mismatch dislocation. This again comes to the question of the true structural nature of the film

4) In terms of the possible stress field as mentioned in 3), then this will affect the Cl ion distribution as well.

Overall, the only new piece of information of this work is the maps of the spatial distribution of chlorine ions. All other characterization and interpretation are not thorough enough to persuade me to believe what they are claiming. I think they need fully characterize the true structural nature of the film, subsequently clarifying the interface mismatch between the film and the matrix, give a good account to the origin of the contrast modulation in the HRTEM image (which is related to the interface mismatch), then I deeply believe they may find they have to re-write the story. The minor point is to correct the improper term of chloride ions.

5) Figure 7 is not self consistent in terms of mass flow, as it is not clear how does this transition happens.

Reviewer #4 (Remarks to the Author):

the authors have addressed a number of my issues - there theoretical analysis is expanded and the reference list updated as well as the minor corrections made

as such the paper is substantially improved

Response to the comments raised by reviewer #3:

We appreciate the general recognition by the reviewer that "the authors take great effort for revising the manuscript, clarifying a lot of confusions", and that "Overall, the work includes some information that may be publishable".

Nevertheless, the reviewer raises some specific concerns especially regarding the structural nature of the passive film. We fully understand the reviewer's concerns, and here we address all these concerns one-by-one in the following.

Concern (1): The authors need to take care regarding the terminology. The term of "chloride ion" is not a correct term, I think they refer to "chlorine ion". So, they need carefully check the term of chloride, chloride ions, chlorine, and chlorine ions. Please make sure to use the right term at the right context, otherwise, it will be misleading.

Response to Concern (1):

We appreciate the concerns raised by the referee. According to the literal interpretation, the term of "chloride ion" is defined as the anion Cl^-, negatively charged ion. It is formed when the atom chlorine (e.g. Cl) gains an electron or when a compound such as hydrogen chloride is dissolved in water or other polar solvents.

Indeed in our present manuscript, we use the term of "chloride ion(s)", referring to the anion "Cl^-" (negatively charged ion). In the meanwhile, we also use the words "chloride", and occasionally use "Cl^- ion" to refer to the same meaning.

Actually, such a wording is frequently used in the field of electrochemistry and corrosion. This can be confirmed by various references in our text numbered 7, 8, 11, 14-21, 23-27, 36, 39, 43 and 46, some of which are well known journals in this field such as "Journal of the Electrochemical Society", "Corrosion Science" as well as "Electrochimica Acta". Meanwhile, the term of "chloride" is often used alone to refer to Cl^- according to the context. In addition, some authors prefer to directly use "Cl^-" or "Cl^- ion(s)" throughout the text. In summary, in expressing the anion Cl^-, chloride ion(s), chloride, Cl^- or Cl^- ions are alternatively used in the field of electrochemistry and corrosion.

Concern (2): The structural nature of the passivation film is not clearly identified. The authors use the term of mostly amorphous, with short range order. This gives an impression of the two phase structure. Scrutinizing at the HRTEM images of Figure 4, one can see the crystal of several nanometers. So the statement of the mostly amorphous structure with short range order is very misleading. I would suggest they use dark field image and electron diffraction pattern to clearly reveal the true structural nature of the film.

HRTEM image can be misleading for interpretation of local structure in relevance to the surrounding environment due to thickness variation caused contrast variation.

Response to Concern (2):

We appreciate the concerns by the referee on the structural nature of the passive film. This reminds us that using the term of "short range order (SRO)" in this case is not accurate, since in the previous version of our manuscript the SROs were used to refer to the tiny crystals with nanometer scale. This deviates the original meaning of SRO in solid state physics. So in this revised manuscript, we use the term of mostly amorphous with some nano-crystals.
Since the crystals in the passive film are very tiny and some of them are not fully crystallized, it is hard to image them by using low-magnification dark-field technique. Nevertheless, based on the reviewer's suggestion, we have also performed the dark-field imaging as appended below, where the contrast of the film is too week to well identify the embedded nano-crystals. In contrast, the nano-crystals are very clearly identified by using high-resolution TEM images, as seen in Figure 4 of the main text and Supplementary Figure 9 of supplementary materials.

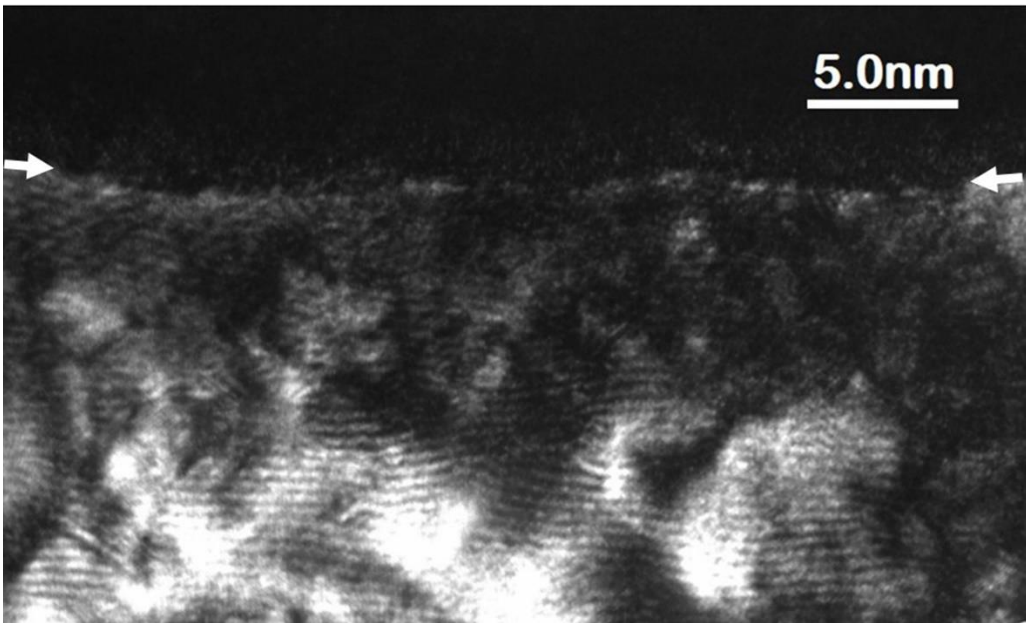

Figure R1: A dark-field TEM image taken at the interface of passive film and matrix. The interface is marked with a pair of head-to-head arrows.

Concern (3): The HRTEM images shows contrast modulations, what the origin of this kind of modulation? is this Moiré fringe (I do not believe that), or something else? Looking into the recent publications on Cu-Au alloy oxidation, it appears similar contrast modulation has been identified to be attributed to the interface mismatch dislocation. This again comes to the question of the true structural nature of the film.

Response to Concern (3):

The alloy of $FeCr_{15}Ni_{15}$ used in the present study is of high plasticity. During the mechanical thinning for electron transparency, the $FeCr_{15}Ni_{15}$ matrix must be deformed with the

presence of dislocations. The dislocations feature a local lattice expansion compared with the neighbouring lattice-perfect area, whose coexistence results in Moiré fringe. This is a general phenomenon for metals as seen in Transmission Electron Microscopy: a text book for materials science (D. B. Williams and C. B. Carter).

It is worthwhile to mention that contrast modulation that the reviewer pointed out only occurs in the $FeCr_{15}Ni_{15}$ matrix (particularly away from the interface) rather than at the film/metal interface, so the contrast modulation in the $FeCr_{15}Ni_{15}$ matrix does not influence our main finding in the passive film and at the interface.

Since the film is mostly amorphous, the interface dislocation, which usually occurs between two crystals, is rare in the present case. Although in some cases the nano-crystals are likely epitaxially grown on the $FeCr_{15}Ni_{15}$ matrix (as that in Supplementary Figure 9 of supplementary materials), they are small enough to accommodate the lattice mismatch.

We would like to emphasize that the remarkable difference of strain state in the matrix side near the metal/passive film interface in Figure 6a and 6b strongly supports the inherence of chloride-induced lattice expansion, since the procedure of TEM specimen preparation for the two different samples is the same.

Concern (4): In terms of the possible stress field as mentioned in 3), then this will affect the Cl ion distribution as well.

Response to Concern (4):

As discussed above, stress field only occurs in the $FeCr_{15}Ni_{15}$ matrix rather than in the film nor at the interface. So, the tress field in the matrix cannot affect the Cl^- ion distribution in the film and at the interface.

Concern (5): Figure 7 is not self consistent in terms of mass flow, as it is not clear how does this transition happens.

Response to Concern (5):

We appreciate the concerns by the reviewer. In describing the interface evolution (Figure 7), we did take into account of the self-consistence for the mass flow, as seen in the illustrations appended below. In figure 7a, the diagrammatic sketches from the left to the right illustrate the four successive steps experienced in passive film formation and growth. The sketch Fig.7a1 shows the bare austenitic matrix experienced selective dissolution of metal ions (major Fe) in a somewhat homogeneous manner; a2 illustrates the initial formation of oxide film wherein the inner barrier layer grows into the metal and the outer precipitation layer is formed via hydrolyzation of the dissolved metal cations. That yields the interface of Me/Film lower than the original metal surface and the outmost surface of the film higher than the original metal surface; a3 illustrates the film further thickens to attain a limit thickness wherein the similar process with a2 continues to occur; a4 shows the interfaces of Me/Film keep moving towards metal when dynamic equilibrium is established, and the thickness of the passive film remained more or less constant. Figure 7b illustrates the same procedure with that in figure 7a, but the rate of oxide growth into metal is heterogeneous induced by chloride yielding an undulating Me/Film interface.

Figure 7c shows the chloride attack on the as-grown passive film which has already attained the dynamic equilibrium.

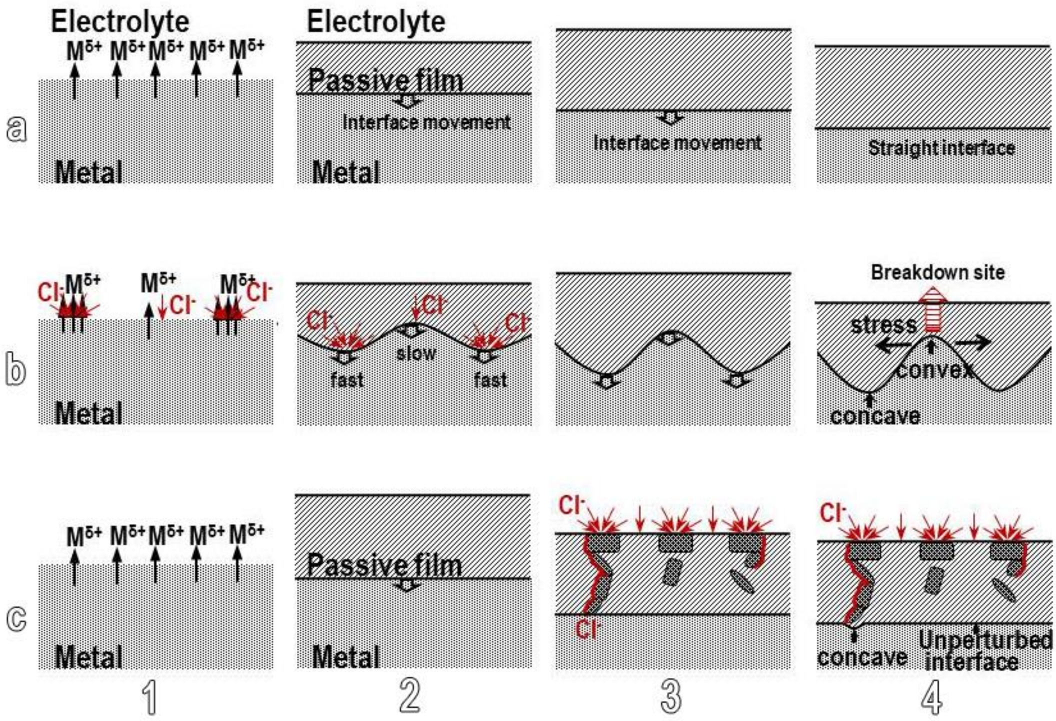

Figure R2 (Figure 7 in the text): Schematic maps illustrating the interface evolution in the absence and presence of chloride ions. The film growth process involves transport of both injected metal ions from the matrix and oxygen in solution through the barrier layer, causing the metal/film (Me/BL) interface to move towards the metal matrix side.
